# Understanding the impact of along-transect resolution on acoustic surveys

**DOI:** 10.1038/s41598-023-40960-6

**Published:** 2023-08-22

**Authors:** Guillermo Boyra, Iosu Paradinas, Iñaki Rico, Udane Martínez, Enrique Nogueira

**Affiliations:** 1 AZTI, Herrera Kaia, Portualdea z/g, 20110 Pasaia, Gipuzkoa Spain; 2 University of St Andrews, St Andrews, KY16 9AJ UK; 3 Centro Oceanográfico de Vigo, Instituto Español de Oceanografía, Subida Radio Faro, 50, 36390 Vigo, Pontevedra Spain

**Keywords:** Acoustics, Ocean sciences, Statistics

## Abstract

Resolution can be defined as the minimum distance between two consecutive sampling points taken by an instrument. In acoustic surveys, the main parameter determining the resolution of sampling along a transect is the distance between successive echosounder transmissions or “pings”. An increase in either the time interval between pings or the speed of the vessel increases the inter-ping distance, hence decreasing the effective sampling resolution. This study investigated whether a loss in along-transect resolution affects the mean backscattered acoustic energy, leading to uncertainty and/or bias in abundance estimates. To this end, a real acoustic survey was echo-integrated, followed by the application of a systematic resampling scheme to simulate a decrease in pinging resolution. For each transect, the mean backscattered acoustic energy calculated at each resolution was compared with that at the original resolution. Transects were characterised according to their heterogeneity and spatial autocorrelation to investigate their effect on the relationship between abundance error and sampling resolution. Uncertainty was seen to increase with decreasing resolution, with higher heterogeneity and lower spatial autocorrelation accelerating the rise in imprecision. Although the mean bias across replicates was zero, the asymmetry of the bias distributions increased with decreasing resolution, leading to an increasing probability and magnitude of underestimation (https://aztigps.shinyapps.io/PingRateStudio/).

## Introduction

Trawl-acoustic methodology is one of the most widely used methods for obtaining abundance estimates of underwater organisms^1–3^. It combines acoustic data collected by active sonar with biological sampling to quantify the density of different fish and nekton species. In trawl-acoustic campaigns, an oceanographic or fishing vessel navigates a portion of the ocean or a lake, describing straight trajectories or transects^4^ until it covers a fraction of the region of interest that is considered representative of the entire area. As the survey progresses, the vessel records acoustic data using a down-looking echosounder, intermittently interrupting the survey to conduct fishing trawls to identify the species present and determine their size distribution. The abundance of fish in a given area, $N$, is proportional to the sum of the acoustic energy backscattered from a layer between two depths in the water column. In acoustic surveys, the most commonly used form of the acoustic backscattering magnitude is ${s}_{A}$, the Nautical Area Scattering Coefficient or NASC^5^ (${m}^{2}{nmi}^{-2}$), conveniently scaled in units of nautical miles to match the units of distance used in maritime navigation. The basic equation used to calculate fish abundance acoustically is as follows^3,6^:

1 $$N=\frac{\langle {s}_{A}\rangle }{\langle {\sigma }_{bs}\rangle }A$$

where $\langle {s}_{A}\rangle$ is the mean of the NASC values measured in the sampled region, $A$ is the area of the entire region of study (${nmi}^{2}$) and $\langle {\sigma }_{bs}\rangle$ is the backscattering cross-cSection^5^ (${m}^{2}$), representing the mean acoustic reflectivity of the fish in the same region. $A$ is often obtained using some form of Geographic Information System; $\langle {\sigma }_{bs}\rangle$ is estimated by applying previously established empirical relationships between acoustic reflectivity and fish length to the species and size distributions obtained from trawl hauls; and $\langle {s}_{A}\rangle$ is obtained by vertically summing and then horizontally averaging the sampled acoustic values. Note that the angle brackets in Eq. (1) (and in the rest of the text) represent the mean value.

**Figure 1:**
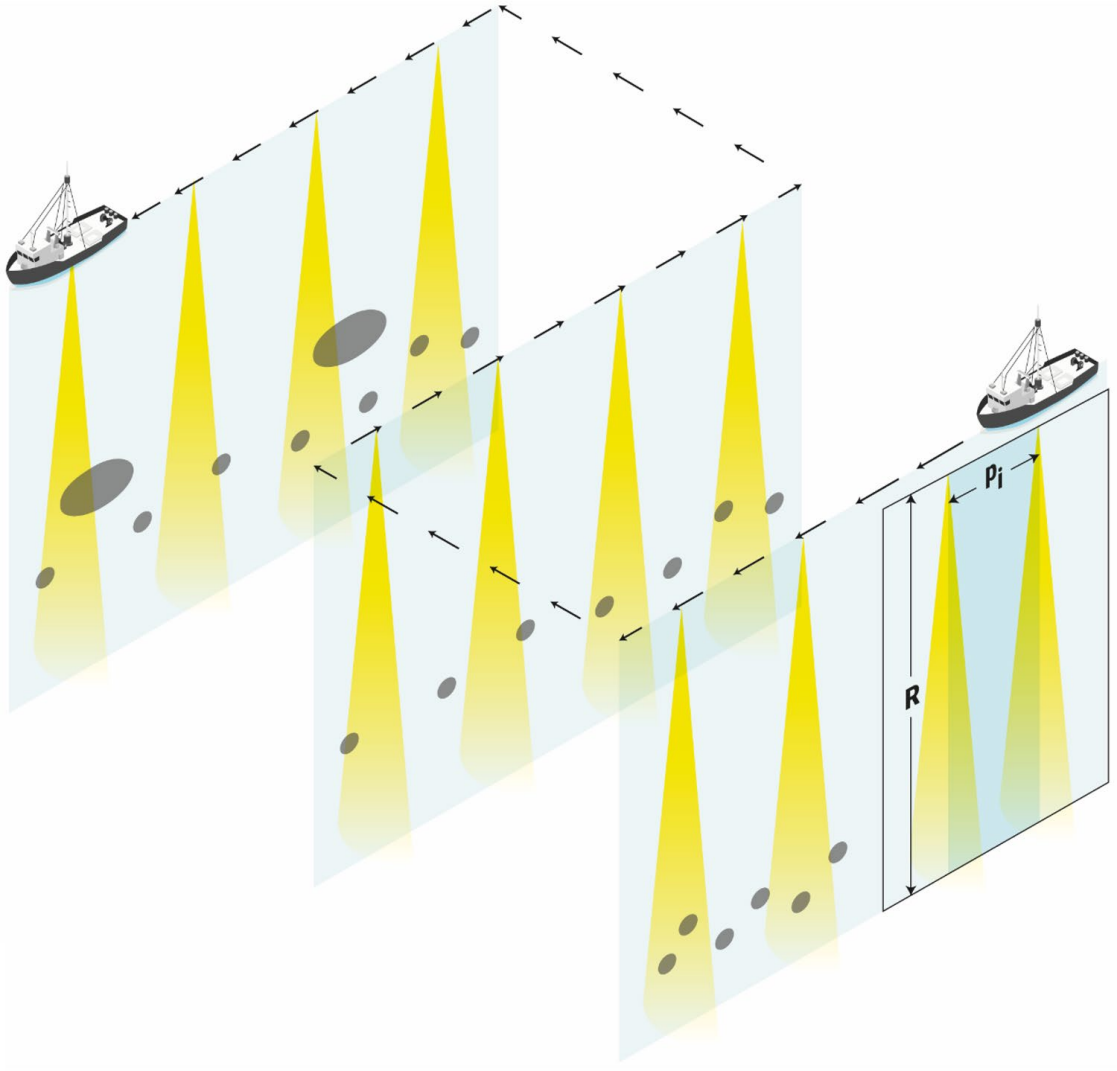
Schematic of the sampling along three transects of an acoustic survey. The yellow triangles represent idealized conical acoustic beams after each transmission or ping, and the grey ellipses represent fish aggregations, both projected onto the along-transect plane. Note that the drawing is not to scale and the distances between transects are usually much greater than the distances between pings.

One of the main advantages of acoustic surveys is that they provide relatively intensive sampling in both the vertical and horizontal directions. For the down-looking echosounders used in trawl-acoustic surveys, sampling in the vertical direction is exhaustive up to the extinction range of the acoustic signal, with a sampling resolution that depends on the sampling rate of the echosounder’s analog-to-digital converter (and, in some systems, also by the averaging interval chosen by the operator), which in narrowband acoustics can vary from millimetres to tens of centimetres depending on the pulse duration and configuration used^3,7^. In contrast, horizontal sampling is not exhaustive and resolution is typically higher along transects than between them. The resolution between transects depends on the sampling design; if the area is covered by parallel transects, it is given by the inter-transect distance (Fig. 1), which is typically on the order of a few kilometres to tens of kilometres^8^. The sampling along the transects is much more intensive, but it is by no means exhaustive, especially in the first layers of the water column, which are partially unsampled due to the approximately conical beam spreading (Fig. 1). The balance between the ping interval (i.e., the time between two consecutive acoustic transmissions or pings) and the speed of the vessel determines the along-transect sampling resolution, measured as the distance between successive pings. While the acoustic pulse length and the inter-transect spacing are normally fixed before the survey starts, the inter-ping distance might be continuously adjusted during the survey (due to changes in the ping interval or vessel speed, as explained below), ranging between 0.5 m and 10 m in most surveys targeting epipelagic species (corresponding respectively to a ping interval of 0.1 s and 2 s for a typical vessel speed of 10 kn, i.e., 5 m/s^8^). In this paper, the focus is only on the along-transect resolution, considering that the other two (vertical and inter-transect) resolutions are fixed.

The change in the vessel speed directly affects how closely the samples are taken for a given ping interval. If the vessel is faster, there will be more distance between consecutive samples, and if it is slower, the samples will be closer together. While the typical trawl-acoustic surveys use vessel speeds around 10 kn, some so-called “surveys of opportunity” use faster fishing or merchant vessels (up to 14 kn or even more^9^) thereby increasing the distance between consecutive pings. On the other hand, autonomous vehicles often move slower, resulting in samples taken closer together.

During acoustic sampling, the ping interval can be modified to accommodate changes in the distance to the seafloor and to avoid aliased seabed echoes (also known as “false bottom” echoes^10^). In addition, the ping interval can be increased for two other important reasons: alternate pinging and increased sampling range. First, alternate pinging involves distributing pings from various sensors into different groups to avoid interference and crosstalk^11^. Recently, it has gained importance due to the commercialization of a generation of wideband echosounders^12^, which has raised the risk of crosstalk due to the enlarged bandwidth of individual sensors. Second, increasing the sampling range increases the ping interval, as it takes longer for the sound to travel a given distance and return. It has also recently gained importance owing to renewed interest in the exploitation of mesopelagic populations and their role in transporting carbon in the ecosystem^13^ following a re-estimation of their abundance^14^. This has led some acoustic surveys previously targeting epi-pelagic species to increase their sampling range from ~ 200 m (the typical maximum depth of the continental shelf) to ~ 1000 m to include mesopelagic species, multiplying the minimum ping distance requirement by a factor of ~ 5 while maintaining the same vessel speed.

One of the main concerns when reducing the sampling intensity is the potential impact that it might have on the acoustic abundance estimates of the target species. A decreased along-transect resolution implies a reduction in the effective number of ${s}_{A}$ measurements that will contribute to the average in Eq. (1). All physical measurements are subject to two types of error: random errors, inherent variability or imprecision of the measurements that cause them to fluctuate around the true value; and systematic errors, persistent offsets that deviate the measurements apart from the true value in one direction, thus leading to bias^15^. According to the Central Limit Theorem, the variance of a sample mean depends on the number of independent samples collected^16^. Therefore, a reduction in sampling resolution should reduce the precision of the mean obtained, thus introducing a random error. It is less clear, however, whether it would also lead to a systematic bias, especially considering the high heterogeneity and tendency towards spatial autocorrelation of fisheries acoustic data^3,17^.

The aim of this study was to analyse whether the loss in along-transect sampling resolution affects the mean acoustic backscattering energy, $\langle {s}_{A}\rangle$, of pelagic species. To this end, a large portion of an acoustic survey was echo-integrated and a systematic resampling scheme was applied to the data to simulate a decrease in the distance between consecutive pings. The uncertainty and bias in $\langle {s}_{A}\rangle$ associated with a reduction in the sampling resolution was assessed. The potential causes of the observed errors were then investigated by building predictive models of random and systematic errors against resolution, accounting for different levels of heterogeneity and spatial autocorrelation of the acoustic data per transect.

## Material and methods

### Data collection

The data analysed were acquired in 2010 during the JUVENA trawl-acoustic survey^18,19^, which targets a variety of small pelagic species (the most important being anchovy, sardine, mackerel, horse mackerel and spratt) in the Bay of Biscay^8^. This particular year was chosen because of the relatively high along-transect resolution used in comparison with the subsequent years when the sampling range was increased to include mesopelagic species among the acoustic targets. Acoustic data covered 1240 km from 54 different transects or transect segments with an average length of ~ 23 km and an average ping interval of ~ 0.5 s (Fig. S1).

Data were collected from the 28 m long research vessel Emma Bardán using downward looking echosounders (Simrad EK60 with 38, 120, and 200 kHz frequencies, with beam angle at  − 3 dB: ~ 7°) mounted on the ship’s hull. In situ on-axis calibration of the echosounders was performed prior to the survey using the standard target methodology^20^. Acoustic sampling was conducted along 30 days in September, during daylight hours (from ~ 05:00 to ~ 19:00 UTC), when fish schools were aggregated in the water column or near the seabed. Acoustic data were recorded with an average ping interval of 0.5 s, vessel speed of 10 kn (5 m/s), and pulse duration of 1 ms (i.e., a vertical resolution of ~ 19 cm), following a systematic sampling design of uniformly spaced (28 km between transects) parallel transects perpendicular to the coast (Fig. S1). The water column was sampled from 10 to 200 m range (see^8,21^ for further details).

### Data analysis

The main objective of the analysis was to measure the influence of sampling resolution on the acoustic-based abundances. As in the trawl-acoustic methodology abundance is estimated using Eq. (1), for simplicity, in this study, the mean NASC was used as a proxy for abundance. The analysis consisted of an echo-integration per cells of the acoustic data (after the removal of the seabed echo and various types of noise) recorded at 38 kHz, with a minimum threshold of  − 60 dB. The size of the cells was 1 ping and 10 m depth, including as many layers as necessary to cover the full vertical extension of the aggregations (total heights ranging from 15 to 100 m with an average height of ~ 50 m). Acoustic echo-integration was performed using Movies + version 4.4 (Ifremer, France).

**Figure 2:**
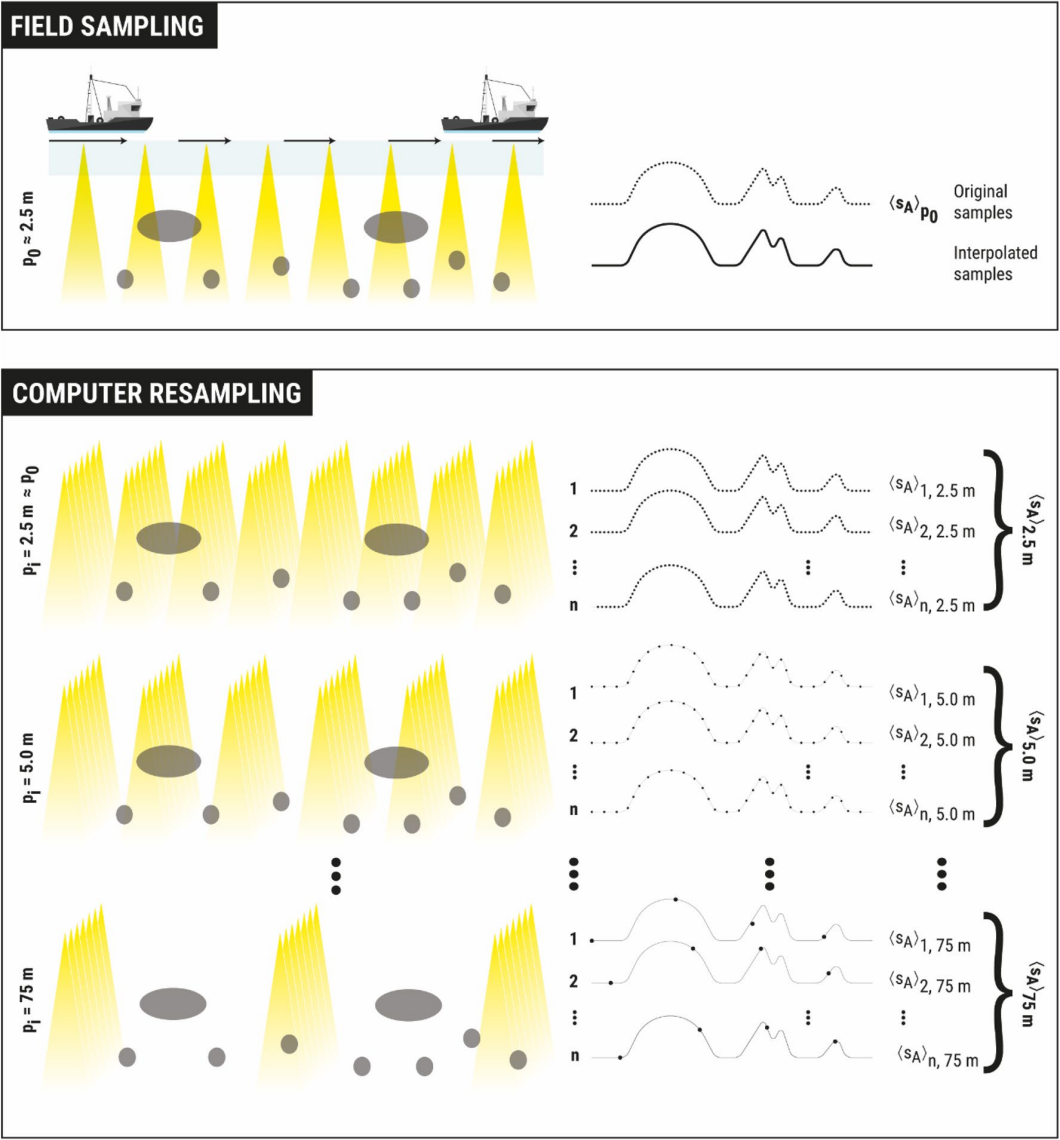
Illustration of the resampling scheme applied to simulate the increase in ping distance (${p}_{i}$) for each transect. The top panel represents the original sampling in an idealized transect, and the lower panel represents a series of successive resampling iterations with decreasing resolution. On the left side, the yellow triangles represent pings and the grey ellipses represent fish aggregations; on the right side, pings are represented by dots, and the eventual bumps formed by the dots represent fish aggregations.

It followed the application of a random series of sequential resampling replicates to simulate a progressive decrease in pinging resolution. The analysis was carried out at the transect level (or transect segment when different parts of a transect were assigned different species compositions according to the echogram scrutiny with the aid of trawl hauls), each considered as an independent measurement. For each transect, the dispersion and bias of the NASC resampled values were then analysed in terms of the simulated ping resolution, as well as the heterogeneity and spatial autocorrelation characteristics of the acoustic data. The basic measure used for ping resolution was the distance between pings or “ping distance”, ${p}_{i}$ (m), although it was also expressed as the equivalent ping interval (measured in seconds), calculated for the mean vessel speed of 10 kn used in the survey. The resampling scheme was applied *n* times to each transect for each ping distance, and then the mean NASC was calculated for each of the *n* sets of data generated at each simulated ping distance to determine the uncertainty and bias (Fig. 2). Resampling and statistical analyses were performed using R^22^.

### Resampling scheme

The key to this work was to realistically simulate the decrease in ping resolution that can occur in an acoustic campaign (i.e., as the sampled range or vessel speed increases) through computer resampling techniques. To achieve this without distorting the underlying spatial structure of the data, systematic resampling was performed within each transect. Systematic (or sequential) means that resampling is carried out in an orderly and chronological manner, with resampling locations homogeneously distributed at constant intervals (Fig. 2). This is intended to mimic the actual acoustic sampling of a real echosounder, which tends to maintain approximately homogeneously spaced ping intervals depending on the survey parameters. The idea was to gradually increase the ping distance to simulate the associated decrease in along-transect resolution. It was therefore assumed that these sequential resamples would preserve the spatial structure of the original data, at least as much as a looser sampling would in the real world. Note that in this study, “sampling” is referred to as the acoustic monitoring originally carried out on the field, and “resampling” is used to indicate the computer-simulated sampling subsequently performed on the original data.

An initial problem faced with this systematic resampling scheme was that, because it consisted of removing some of the available pings, the possible combination of pings to be removed varied with the resampling resolution. As a result, for some resolutions, there were not enough repetitions, resulting in resampling sets of unbalanced size, making it difficult to determine whether some of the observed uncertainty was due to the loss of sampling resolution per se or to the lack of sufficient repetitions. To overcome this problem, a linear interpolation of the original data was performed within each transect (using the "approach" function of R), which allowed to perform systematic resampling using any possible ping distance. In order to obtain a sufficiently large number of samples from which to make statistical inferences, random transect origin delays (up to 30 s) were applied in each replicate to achieve variability, and 3000 resampling repetitions were performed at each ping distance (Fig. 2). Using this resampling scheme, a progressive sequence of simulated ping resolution was obtained from the minimum of the original mean resolution of 2.5 m (corresponding to a ping interval of 0.5 s, given the vessel speed of 10 kn in the original acoustic survey) to a maximum of 75 m in 2.5 m increments (Fig. 2).

Each replicate of systematic resampling resulted in a measure of mean acoustic density per transect. These sets of 3000 mean values per transect and ping distance were then treated as bootstrap samples^23^. The resulting resampling distributions were examined by constructing statistical metrics to infer the average precision and bias for each transect and for every ping distance in the sequence.

### Estimation of abundances per transect

While acoustic backscatter was echo-integrated at the highest available along-transect resolution (1 ping), echo-integration in acoustic surveys is usually performed using lower along-transect resolution cells, defined by the Elementary Distance Sampling Unit (EDSU), which is often chosen as 1 nmi (1852 m^24^). Therefore, to realistically simulate an acoustic survey, the mean abundance per transect was estimated in two steps: first, the mean NASC values were calculated for each of the 1-nmi EDSUs, and then they were averaged over the transects. The 1-ping NASCs were used to simulate the reduction in resolution, and the intermediate 1-nmi NASC means were used to obtain results comparable to those of typical surveys.

The mean NASC for the original ping interval, ${\langle {s}_{A}\rangle }_{{p}_{0}, e, t}$, was calculated as an average over the pings in each EDSU to be used later as a reference for bias calculations:

2 $${\langle {s}_{A}\rangle }_{{p}_{0}, e, t}= \frac{1}{{N}_{e,{p}_{0}}}\sum_{j=1}^{{N}_{{e,p}_{0}}}{s}_{{A}_{j, {p}_{0},e,t}}$$

where $j$ represents each ping, ${p}_{0}$ is the original ping resolution (~ 2.5 m), $e$ is the EDSU ($n.mi.$), $t$ is the transect, ${N}_{e,{p}_{0}}$ is the number of pings in the EDSU sampled at the original ping distance and ${s}_{{A}_{j,{p}_{0},e, t}}$ is the NASC of each ping in the EDSU. The mean NASC for the original ping interval in each transect, ${\langle {s}_{A}\rangle }_{{p}_{0},t}$, was then calculated averaging ${\langle {s}_{A}\rangle }_{{p}_{0}, e, t}$ over the EDSUs in the transect:

3 $${\langle {s}_{A}\rangle }_{{p}_{0}, t}= \frac{1}{{N}_{t}}\sum_{e=1}^{{N}_{t}}{\langle {s}_{A}\rangle }_{{p}_{0}, e, t}$$

where ${N}_{t}$ is the number of EDSUs in the transect and $e$ is the EDSU.

Similarly to Eq. (2), when each transect was systematically resampled at different ping distances, ${\langle {s}_{A}\rangle }_{{p}_{i}, e, t,r}$, the mean NASC per EDSU (*e*) and transect (*t*) was also obtained for each ping distance (${p}_{i}$) in each replicate (*r*):

4 $${\langle {s}_{A}\rangle }_{{p}_{i}, e, t,r}= \frac{1}{{N}_{e,{p}_{i}}}\sum_{j=1}^{{N}_{{e,p}_{i}}}{s}_{{A}_{j, {p}_{i},e,t,r}}$$

where ${N}_{e,{p}_{i}}$ is the number of pings per EDSU at a given ping resolution and ${s}_{{A}_{j,{p}_{i},e, t,r}}$ is the NASC of each ping in the EDSU. Mean NASCs, ${\langle {s}_{A}\rangle }_{{p}_{i},t, r}$, per transect (*t*) for each ping resolution (${p}_{i}$) and repetition (*r*) were then obtained by averaging ${\langle {s}_{A}\rangle }_{{p}_{i}, e, t,r}$ over the EDSUs of the transect:

5 $${\langle {s}_{A}\rangle }_{{p}_{i}, t, r}= \frac{1}{{N}_{t}}\sum_{e=1}^{{N}_{t}}{\langle {s}_{A}\rangle }_{{p}_{i}, e, t,r}$$

where ${N}_{t}$ denotes the number of EDSUs in the transect. These computed ${\langle {s}_{A}\rangle }_{{p}_{i}, t, r}$ values are symbolically represented for each resampled repetition in an idealized transect in Fig. 2.

The mean NASC per transect (*t*) and ping resolution, ${\langle {s}_{A}\rangle }_{{p}_{i}, t}$, was calculated by averaging over the bootstrapped repetitions:

6 $${\langle {s}_{A}\rangle }_{{p}_{i}, t}= \frac{1}{n}\sum_{r=1}^{n}{\langle {s}_{A}\rangle }_{{p}_{i},t,r}$$

where *r* represents each resampling repetition, $n$ is the number of repetitions, ${p}_{i}$ is the simulated ping resolution and ${\langle {s}_{A}\rangle }_{{p}_{i}, t,r}$ is the mean NASC of each transect, ping resolution and repetition.

### Analysis of abundance estimation errors

A central assumption in this study is that the abundance estimated with the original pinging resolution of 2.5 m was correct. Two types of error were examined for each transect and ping resolution: (1) the dispersion of the distribution of resampled abundances and (2) the deviation of these resampled abundances from those obtained with the original ping interval (assumed true). The dispersion, ${s}_{{p}_{i}, t}^{2}$ ${(\left({m}^{2}{nmi}^{-2}\right)}^{2}$), of the resampled data was estimated using a standard statistical formulation. It was based on the variance of each transect and ping resolution, estimated as the sum of the squares of the bootstrapped residuals of the mean acoustic density:

7 $${s}_{{p}_{i}, t}^{2}= \frac{1}{n-1}\sum_{r=1}^{n}{\left({\langle {s}_{A}\rangle }_{{p}_{i}, t, r}-{\langle {s}_{A}\rangle }_{{p}_{i}, t}\right)}^{2}$$

where $n$ is the number of repetitions, ${\langle {s}_{A}\rangle }_{{p}_{i},t, r}$ is the mean NASC per transect (*t*) for each ping resolution (${p}_{i}$) and repetition (*r*), and ${\langle {s}_{A}\rangle }_{{p}_{i}, t}$ is the mean NASC per transect and ping resolution. The standard deviation per ping resolution and transect, ${s}_{{p}_{i},t}$ (${m}^{2}{nmi}^{-2}$), was obtained as the squared root of the variance (${s}_{{p}_{i}, t}^{2}$):

8 $${s}_{{p}_{i},t}= \sqrt{{s}_{{p}_{i}, t}^{2}}$$

Finally, the coefficient of variation per transect, ${CV}_{{p}_{i},t}$ (%), was defined as:

9 $${CV}_{{p}_{i},t}=100\frac{{s}_{{p}_{i},t}}{{\langle {s}_{A}\rangle }_{{p}_{i},t}}$$

where ${s}_{{p}_{i},t}$ is the standard deviation per ping resolution and transect, and ${\langle {s}_{A}\rangle }_{{p}_{i},t}$ is the mean NASC per transect (*t*) and ping resolution. To study bias, the deviation, ${\langle Dev\rangle }_{{p}_{i},t,r}$ (%), of the mean resampled NASC per transect from that of the original ping resolution was calculated for each transect (*t*), ping resolution (${p}_{i}$) and repetition (*r*) as follows:

10 $${\langle Dev\rangle }_{{p}_{i},t,r}=100\left(\frac{{\langle {s}_{A}\rangle }_{{p}_{i},t,r}}{{\langle {s}_{A}\rangle }_{{p}_{0}, t}}-1\right)$$

where ${\langle {s}_{A}\rangle }_{{p}_{i}, t, r}$ is the mean NASC per transect (*t*) for each ping resolution (${p}_{i}$) and repetition (*r*), and ${\langle {s}_{A}\rangle }_{{p}_{0}, t}$ is the mean NASC for the original ping interval. These sets of 3000 deviation values per transect and ping distance (also referred to as bootstrap samples) were plotted to visualise their dispersion, central tendency and skewness level for different ping distance values. In addition, different statistics were applied to the distributions of the bootstrap deviation samples, such as the mean, median, mode and probability of underestimation, in an attempt to summarise and quantitatively analyse the evolution of bias with decreasing ping resolution. Three main summarising statistic descriptors were obtained for bias: the mean and modal deviation, and the probability of underestimation. The mean deviation per transect and ping resolution was calculated by averaging over bootstrapped replicates:

11 $${\langle Dev\rangle }_{{p}_{i},t}=\frac{1}{n}\sum_{r=1}^{n}{\langle Dev\rangle }_{{p}_{i},t,r}$$

where ${\langle Dev\rangle }_{{p}_{i},t,r}$ is the deviation of the mean resampled NASC per transect, *r* is the replicate and *n* is the number of replicates. The modal deviation was the most frequent value of each distribution, and the probability of underestimation was calculated as the ratio of the number of negative deviations to the total number of bootstrapped replicates for each transect and ping resolution.

Finally, to simulate the effect of EDSU lengths different than 1 nmi on the error calculations, the bias was assumed invariant and, according to the Central Limit Theorem, the variance levels were assumed to be proportional to the number of samples, hence estimating the change in ${CV}_{e}$ at any given EDSU as proportional to the fraction of the number of samples:

12 $${CV}_{e}=\sqrt{\frac{{N}_{{e}_{0}}}{{N}_{e}}}{CV}_{{e}_{0}}\cong \frac{1}{\sqrt{{L}_{e}}}{CV}_{{e}_{0}}$$

where $${CV}_{{e}_{0}}$$ is the coefficient of variation at a 1 nmi EDSU, $${N}_{e}$$ and $${N}_{{e}_{0}}$$ are the number of pings of a given EDSU ($$e)$$ and a 1 nmi EDSU ($${e}_{0}$$) respectively, and $${L}_{e}$$ was the length of the EDSU in nautical miles.

### Characterization of acoustic data

To help explain the variation in abundance dispersion and bias with sampling resolution, a set of characteristics of the acoustic data per transect were selected. Mean acoustic characteristics per transect were calculated in three broad groups that were considered to have a potential impact in abundance accuracy: fish aggregation typology, heterogeneity and spatial structure.

#### Heterogeneity

Heterogeneity is one of the main characteristics of fisheries acoustic data, which are known to consist of many small observations and a few extremely large ones^25^. Heterogeneity was chosen to represent the extreme value content of each transect, because of the importance of these values, which was reflected in a preliminary analysis of the data (Fig. S2). The high heterogeneity of the acoustic data is reflected in the typically skewed distributions (Fig. S3) where the means are largely driven by the extreme observations, making them particularly unreliable for central estimates.

To define the level of heterogeneity of each transect, different “inequality” indices were calculated, such as Gini, Standard deviation, Entropy, Theil, Atkinson and RS^26^, available in the R package “ineq”^27^. The indices were calculated at the 1 nmi EDSU resolution level, to facilitate comparison with typical trawl-acoustic survey data. In earlier versions of this analysis, all of these indices were used as alternative heterogeneity descriptors, but as they all were found to be highly correlated (Fig. S4), this study focused on the Gini index due to its intuitive interpretation based on the Lorenz curve (as explained below). However, the standard deviation was also included as an alternative, better known and easier to calculate heterogeneity index in the shiny application, provided as a complementary interactive prediction plot.

The Gini index is a measure borrowed from economics, where it is used to rank groups of people (usually populations of countries) according to inequality of their income distribution. The Gini index is bounded between 0 and 1, with a value of 0 for perfect equality and a value of 1 for perfect inequality. The index is based on the Lorenz curve (Fig. S5), a graphic constructed by aligning all the items (in our case pings) in a group (transect) with respect to an increasing characteristic expressed in relative terms (here, the NASC), and then plotting their cumulative distribution (Fig. S5). The Gini index is then calculated as:

13 $$Gini= \left({A}_{T}-{A}_{L}\right)/{A}_{T}$$

where ${A}_{T}$ is the area of the “perfect equality” triangle and ${A}_{L}$ is the area under the Lorenz curve. When the Lorenz curve approaches the diagonal of the triangle, the area under the curve is similar to the area of the triangle and the Gini values are close to 0; whereas when the area under the curve is very small, the Gini values are close to 1 (Fig. S5).

For each transect, the heterogeneity values were calculated at the original, maximum ping resolution, and then averaged per EDSU of 1 nmi. However, as heterogeneity is likely to change its value with resolution, the Gini values were also calculated for all ping resolution distances in the sequence. This was done to study the evolution of heterogeneity with the ping resolution and to infer, in the case of calculating a Gini for a lower sampling resolution, what the equivalent Gini would have been if calculated at the 2.5 m resolution used in this study.

#### Spatial autocorrelation

Spatial autocorrelation refers to the degree of contagion between nearby data values, and was analysed using geostatistical techniques^28^. Geostatistical analysis is based on the semi-variogram of the data (variogram for short), which is used to plot the variability between data points as a function of their mutual distance (Fig. S6). A number of features are extracted from each variogram to describe the spatial autocorrelation structure of the data. The range is the distance at which the model flattens out, that is, at which the observations become independent. It is interpreted as the radius of the spatial structure, the limit beyond which there is no autocorrelation. The sill is the spatially structured part of the variance, and the nugget (the value at which the variogram intersects the y-axis) can be interpreted as the part of the variability that is not explained by the autocorrelation.

Sample variograms (Fig. S7) were derived empirically from the data using the R *gstat* package^29^. The modelled variograms were first fitted by numerical optimisation and then checked and corrected by visual inspection where necessary. The model fit provided variogram features (nugget, sill and range) for each transect (Fig. S6). A proxy for the spatial autocorrelation level was then estimated from these features. The estimated variance of the mean depends on both the balance between sill and nugget and the balance between the correlation range and the region dimension^30^. If the percentage of structured variance (the sill) is low and if the correlation range is small compared with the region dimension, the estimation variance is expected to be large. Conversely, a large sill and correlation range should result in low variance. Consequently, a proxy for the spatial autocorrelation structure, $Corr$, was defined as the product of the two proportions:

14 $$Corr= {P}_{sill}\cdot {P}_{range}$$

where the sill proportion, ${P}_{sill}$, was calculated as:

15 $${P}_{sill}= sill/\left(sill+nugget\right)$$

and the correlation range proportion as:

16 $${P}_{range}= range/(range+ L)$$

where $L$ is the transect length.

#### Fish aggregation typologies

A “school processing” (with parameters detailed in Table S1^31^) was applied to the acoustic backscattering echograms, using Movies + version 4.4, to obtain fish aggregation typologies^32^. The school processing consists of an image segmentation process applied to the echograms to detect and isolate the echotraces corresponding to each individual aggregation to obtain its morphological characteristics^33–35^. Average aggregation characteristics per transect were then obtained (Table S2). After removing the most correlated variables (based on Variance Inflation Factor analysis) and filtering the most significant ones, the selected variables were: mean aggregation NASC and area, mean distance between aggregations (Dist) and occupation rate (OccRate), i.e., the proportion of transect pings occupied by schools.

### Relationship between error and ping interval

To explore uncertainty and bias, scatterplots of mean NASC and deviation per transect against ping resolution (both as ping distance and equivalent ping interval) were constructed, using the different characteristics of the acoustic data in each transect as auxiliary variables. However, as preliminary results indicated that morphological aggregation characteristics provided less explanatory power than heterogeneity and spatial autocorrelation (Table S3), and in an attempt to simplify the communication of the main results of the study, the former variables were not explored further, and the remainder of the paper focuses on the latter two.

Bootstrapped distributions of resampled deviation values were plotted for each ping resolution and heterogeneity and correlation level. Several statistical descriptors were then provided to summarise the bootstrapped distributions. Uncertainty CV, mean and modal deviation, as well as the probability of underestimation were used to summarise the resulting deviation distributions and were plotted against ping resolution. Linear and non-linear (GAM^36^) regression models were fitted to the scatterplots to obtain predictions of the statistical descriptors as a function of ping resolution, with heterogeneity and autocorrelation as auxiliary variables.

To facilitate the interpretation of the analysis, heterogeneity (Gini) and spatial autocorrelation (Corr) were grouped into three bins, each containing 4–6 transects, and were included in the regression models as categorical variables. The categorical bins for heterogeneity were constructed as follows: low–Gini ≤ 0.4; mid–0.4 < Gini ≤ 0.6; high–Gini > 0.6. And the bins for spatial autocorrelation were as follows: low–Corr ≤ 0.33; mid–0.33 < Corr ≤ 0.66; high–Corr > 0.66. Linear and GAM models were compared using the Akaike Information Criteria (AIC).

An interactive application based on Shiny (https://shiny.rstudio.com/) was developed to incorporate the main model results of the resampling exercise and provide more flexible predictions. The application allowed to increase the level of detail and flexibility of the plots, adding the possibility to switch between two of the available heterogeneity indices and to consider EDSU values other than 1 nmi.

**Figure 3:**
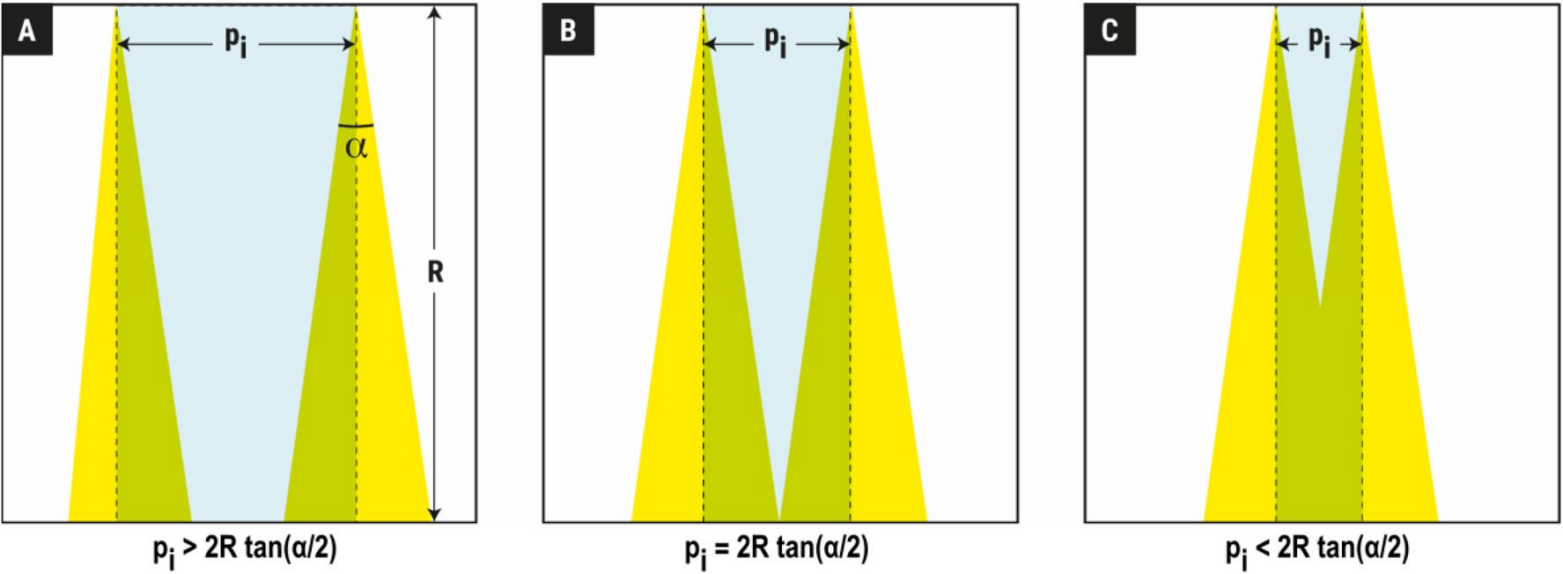
Illustration of the difference in the fraction of the area of the along-transect plane that is sampled, depending on the distance between two contiguous pings (${p}_{i}$), the maximum range ($R$) and the transducer beamwidth ($\alpha$) according to Eq. (17). In (**A**) the sampling resolution is low, and there are unsampled areas in all ranges. In (**B**) the diameter of the ideal beam cone is equal to the distance between contiguous pings. Beyond this point, as ${p}_{i}$ decreases (**C**), there is increasing overlap between contiguous pings, and the sampled fraction increases, with the unsampled areas confined to the upper layers of the water column.

Finally, in an attempt to provide a more comprehensive metric of along-transect resolution, the sampled fraction (${f}_{s}$), that is, the fraction of the transect plane (i.e., the plane defined by the along-transect and the vertical directions; Fig. 3) that was actually sampled was calculated as follows:

17 $${f}_{s}=\left\{\begin{array}{c}\frac{R\, \mathrm{tan}(\alpha /2)}{{p}_{i}}, if\, {p}_{i}\ge 2R\, tan(\alpha /2)\\ 1-\frac{{p}_{i}}{4R\,\mathrm{tan}\left(\alpha /2\right)}, if\, {p}_{i}<2R\, tan(\alpha /2)\end{array}\right.$$

where ${p}_{i} is$ the ping distance, $R$ is the maximum sampling range and $\alpha$ is the transducer beamwidth. This metric was used to provide qualitative insight about the potential effect of different transducer beamwidths on the studied relationships.

## Results

**Figure 4:**
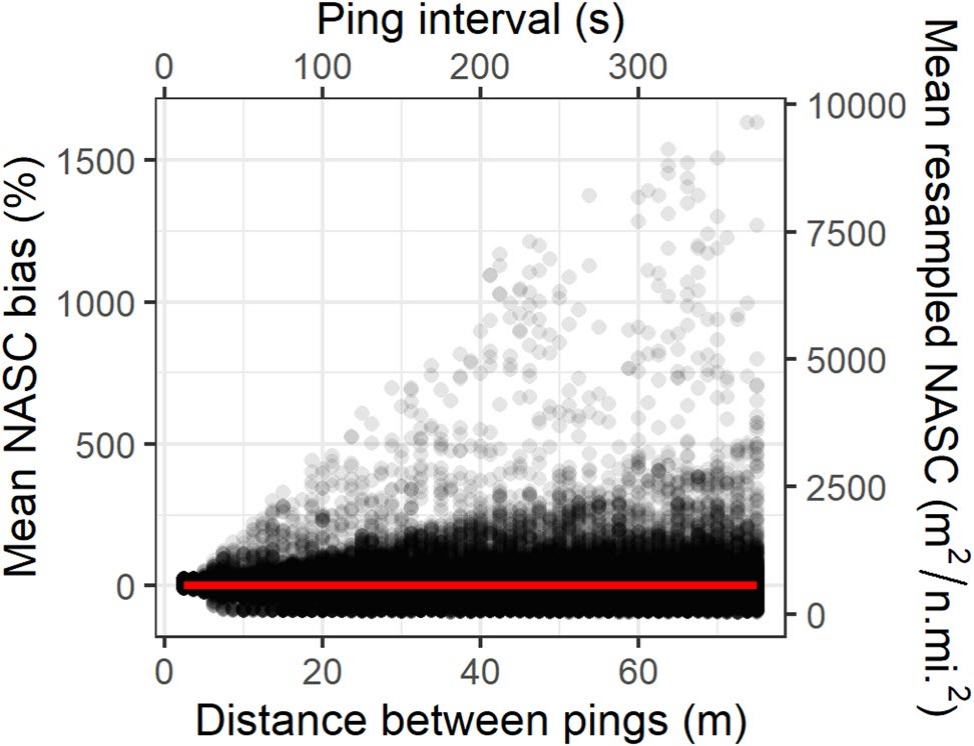
Scatterplot of mean resampled NASC values, ${\langle {s}_{A}\rangle }_{{p}_{i}, t, r}$, and mean NASC bias, ${\langle Dev\rangle }_{{p}_{i},t,r}$, per transect and repetition as a function of ping distance for the resampling exercise. Each dot represents a single repetition of mean NASC and deviation value per transect. Dots have been allowed certain degree of transparency to highlight areas with different density of overplotting. The trumpet shape reveals the increase of uncertainty with increasing ping interval, i.e., decreasing sampling resolution. The continuous line (red in the online version) marks the fitted linear model with zero slope, meaning that after many repetitions the mean bias is zero despite of the increased uncertainty. The equivalent ping interval was estimated using constant vessel speed of 5 m/s (i.e., ~ 10 kn).

The scatterplot of the mean resampled NASC values against the simulated ping interval showed some revealing features (Fig. 4): (1) the variability of the mean NASC values increased with the ping resolution; (2) a marked asymmetry was observed in this increase, the mean resampled NASC values reached higher overestimated than underestimated differences with respect to the original mean values per ping resolution; (3) considerably higher density of underestimated than overestimated values; and (4) as a consequence of the latter two features, the mean bias was zero over the whole range of ping intervals because the more frequent and slightly underestimated mean abundances were compensated by the rare but strongly overestimated ones.

**Figure 5:**
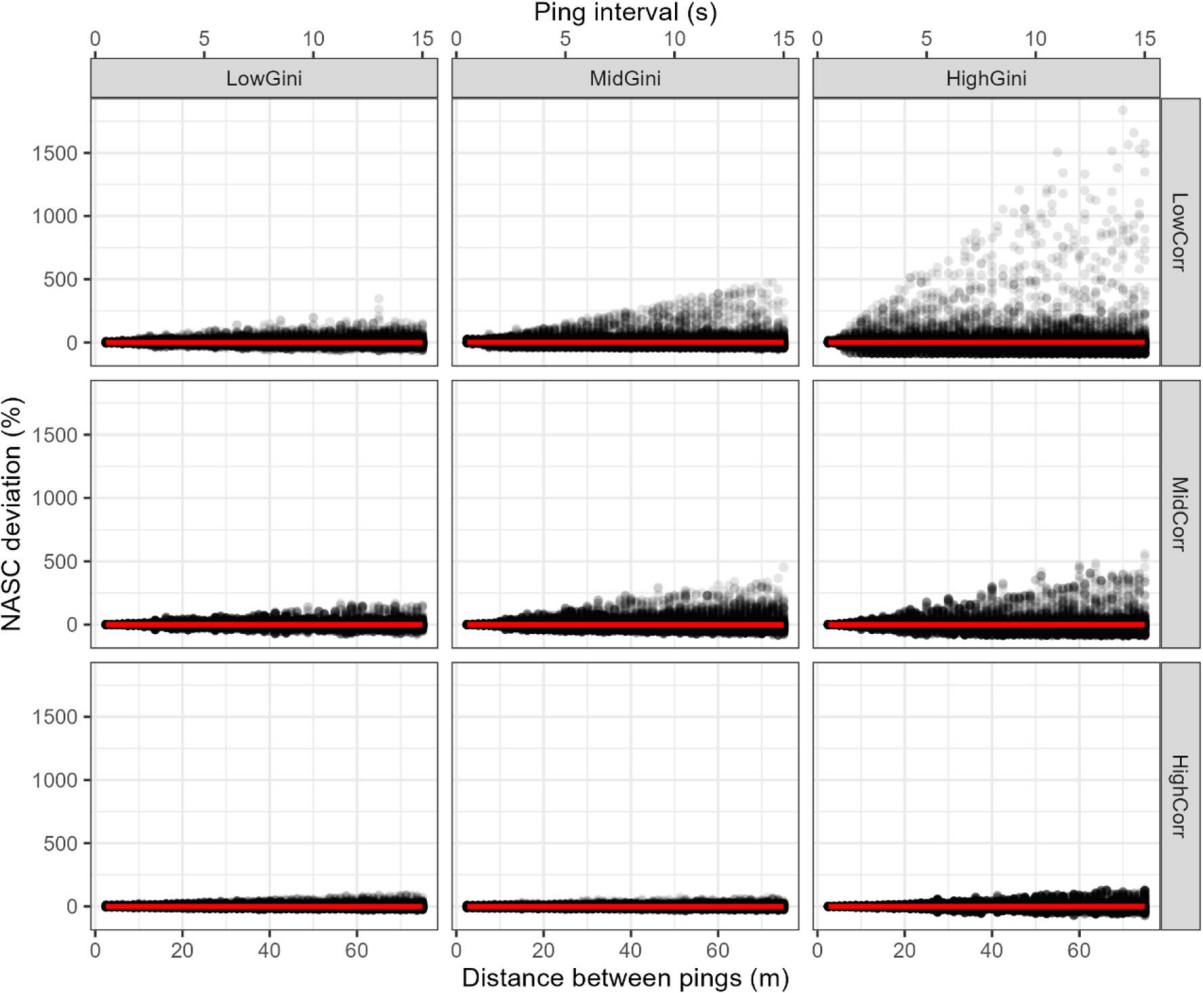
Scatterplots of mean NASC deviation per transect, ${\langle Dev\rangle }_{{p}_{i},t,r}$, against ping distance for different levels of heterogeneity (Gini index values, in columns) and spatial autocorrelation (in rows) of the acoustic data. Each dot represents a single repetition of mean NASC value per transect. Dots have been allowed certain degree of transparency to highlight areas with different density of overplotting. The fitted linear regression models (solid lines, red online) show a flat zero-bias tendency for all heterogeneity and autocorrelation values.

When the scatterplots were segregated by level of heterogeneity and spatial structure, new patterns emerged. The NASC deviation values against ping resolution were observed to increase faster with higher heterogeneity (high Gini values) and lower spatial autocorrelation levels (Fig. 5), being this increase slightly faster increase with autocorrelation. Linear smoothers revealed zero bias regardless of the level of heterogeneity and spatial structure.

**Figure 6:**
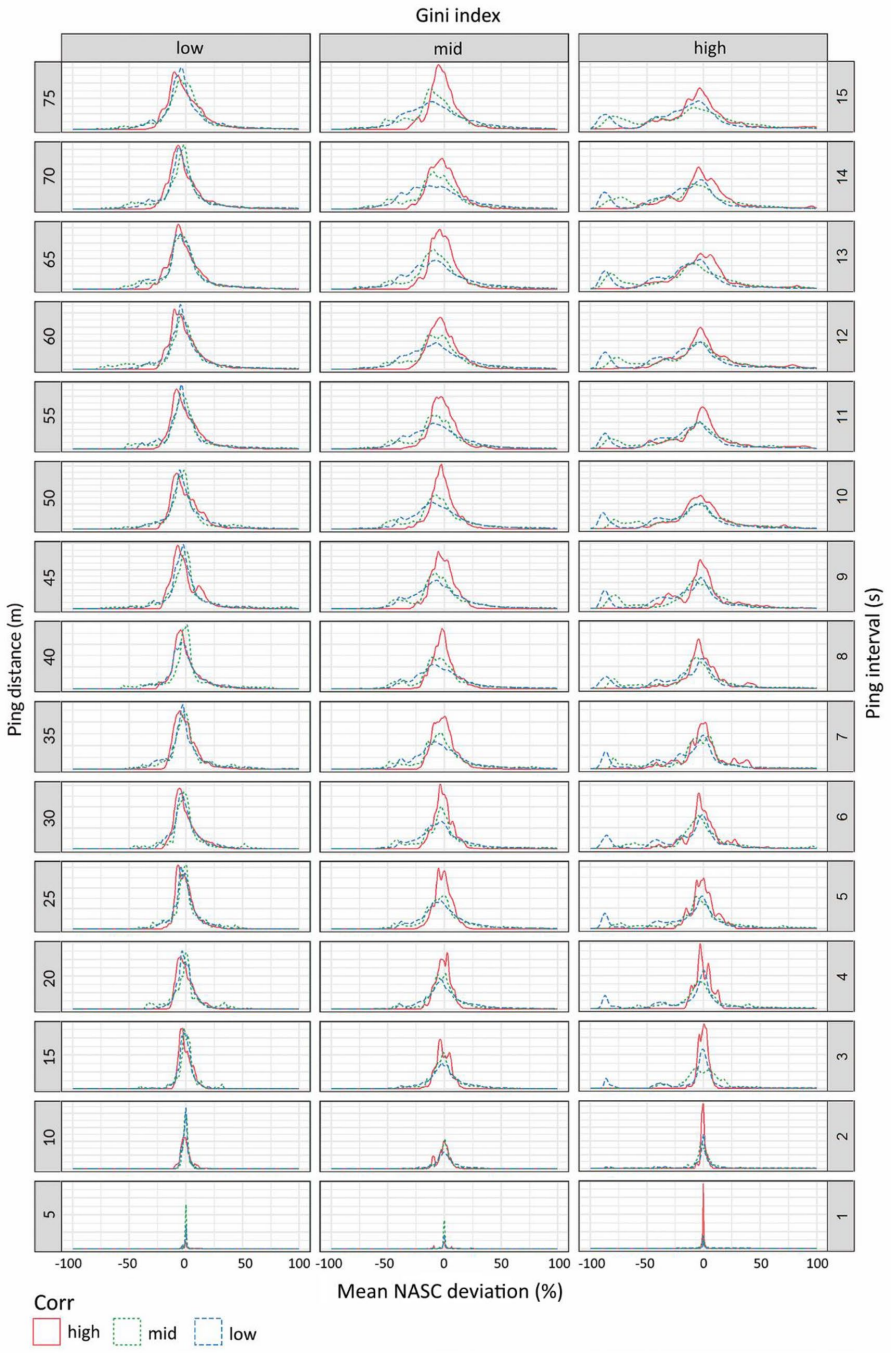
Density plots of the bootstrapped distributions of mean NASC deviation per transect, ${\langle Dev\rangle }_{{p}_{i},t,r}$. Panels represent different heterogeneity levels (Gini index values, different columns), ping distances (in different rows, the number showing the ping distance in m) and spatial structure levels (in different line types and colours). Notice that the upper limit of the horizontal axes are truncated at 100% to improve the visibility of the patterns.

**Figure 7:**
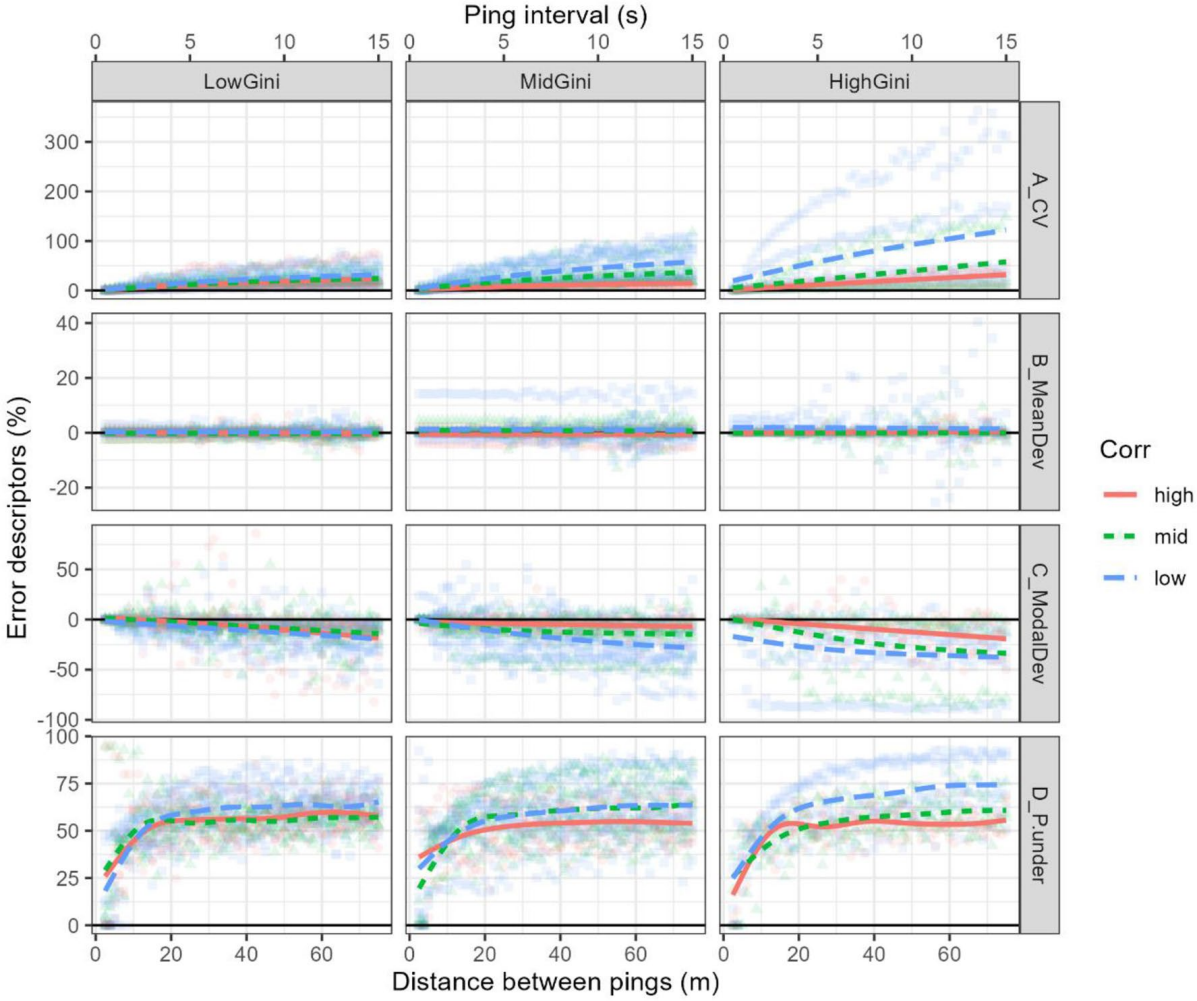
Uncertainty CV (**A**), mean (**B**) and modal (**C**) deviation and probability of underestimation (**D**) (all in %) are plotted against ping distance, for different heterogeneity levels (Gini index values, in the different columns) and different spatial structure levels (in different colors and line types). The lines mark the fitted GAM smoothers, represented to highlight the tendencies. An interactive version of this figure can be accessed through the following link: https://aztigps.shinyapps.io/PingRateStudio/.

The bootstrapped distributions of the deviation values for different heterogeneity levels and ping resolutions (Fig. 6) support a similar view. The bell-shaped curves widened with decreasing ping resolution, and they widened faster with higher heterogeneity. At low Gini levels, the spatial autocorrelation structure did not have much effect, but at higher Gini levels, high spatial structures slowed the increase in distribution width. The bootstrapped distributions also revealed an increasing tendency towards asymmetry with decreasing resolution, leading to more frequent underestimation, especially for high heterogeneity and low spatial autocorrelation. For moderate distances between pings (less than 15 m, corresponding to a 3 s ping interval), the bias was usually close to zero, even for high data heterogeneity and low spatial structure. As the distance between pings increased, the combination of high heterogeneity and low autocorrelation led to a high probability of underestimating abundance. In fact, the bias distribution showed a secondary peak at  − 80%, that is, there was a considerable probability of missing 80% of the biomass in some low-resolution cases. For low levels of heterogeneity, the width of the bias distributions increased at low ping resolutions but remained relatively symmetric regardless of the ping interval and level of spatial correlation. The error descriptors used to summarise the bootstrapped bias distributions showed similar but more quantitative effects of the autocorrelation and heterogeneity on ping resolution (Fig. 7, Table S3).

Concerning the different heterogeneity indices shown in the interactive application provided as an alternative to Fig. 7, both the standard deviation and the Gini index had similar effects in terms of increasing the rate of increase of uncertainty and modal deviation with ping distance (https://aztigps.shinyapps.io/PingRateStudio/). Increasing the EDSU values reduced the uncertainty levels for any sampling resolution.

Finally, the probability of underestimation exhibited a particular pattern with the ping resolution. For small ping distances, the probability of underestimation was low and increased with the ping distance, reaching 50% at ~ 12.5 m and stabilising at ~ 30 m (Fig. 7). This maximum probability of underestimation reaching stability was fairly insensitive to heterogeneity but increased inversely to autocorrelation levels. Additionally, an inter-ping distance of less than 12.5 m (corresponding to a 2.5 s ping interval at 10 kn) appeared to be relatively safe for typical acoustic surveys in terms of low expected probability and levels of underestimation.

The decrease in AIC when covariates were added demonstrated their usefulness in capturing the change in uncertainty with ping resolution. Both the heterogeneity and spatial structure were significant covariates (Table S3). In terms of linearity, GAMs provided slightly but consistently lower AIC values than linear models for all statistical descriptors.

## Discussion

### Effect of ping resolution on acoustic density estimation error

The main aim of this study was to answer a key question about the importance of sampling intensity: Can low-resolution acoustic sampling lead to loss of precision, bias, or both when estimating the abundance of a given population? In terms of precision, the resampling exercise showed that, as expected, the dispersion of acoustic abundances increased as the sampling resolution was reduced (Fig. 4). This is consistent with the Central Limit Theorem^16^, which predicts an increase in variance as the number of independent samples used for estimation decreases. This result is also in qualitative agreement with previous empirical results in acoustic surveys^37–40^, where precision was found to be positively correlated with the fraction of volume effectively sampled. However, these previous papers focused on resolution across transects, with along-transect resolution considered to be continuous. Here, it is shown that the resolution along the transect affects the uncertainty of the acoustic abundances (Fig. 4), not only because it is not exhaustive but also because the fraction of the transect plane acoustically sampled decreases with ping distance (Fig. 1).

Another novelty of this study is that, in addition to analysing uncertainties in the estimates (as did the empirical studies mentioned above), it also analyses bias, i.e. potential systematic deviations from the true abundance. To deepen in the understanding of the estimation bias, the bootstrapped distributions of NASC deviations (Fig. 6) were summarised using different statistical descriptors (Fig. 7). While the uncertainty was relatively straightforward and could be analysed using a single descriptor (CV), the study of bias was more complex and required three different descriptors (mean deviation, modal deviation and probability of underestimation) to disclose it. The observed mean deviation was zero regardless of the ping resolution, showing that the result was strictly unbiased (note the flat smoothers in Figs. 4,  5 and  7B). Of course, this was also expected as a result of the Weak Law of Large Numbers, according to which, as the sample size increases, its mean approaches the population mean^41^. Therefore, in our case, after many repeated resamples of each transect, it was not surprising to obtain resampled means close to the original ones (i.e., unbiased).

More interestingly, however, despite the expected overall unbiasedness, there were consistent asymmetric trends in the variability (Fig. 6) that led to likely underestimation in practice, as revealed by the modal deviation and probability of underestimation (Fig. 7C and  D). On the one hand, the modal (i.e., the most likely) deviation from the true value increased with ping distance. On the other hand, the probability of underestimation was lower than that of overestimation at small ping distances, increased with increasing ping distances (exceeding that of overestimation for intervals above ~ 12.5 m), and reached stability at 25–35 m.

**Figure 8:**
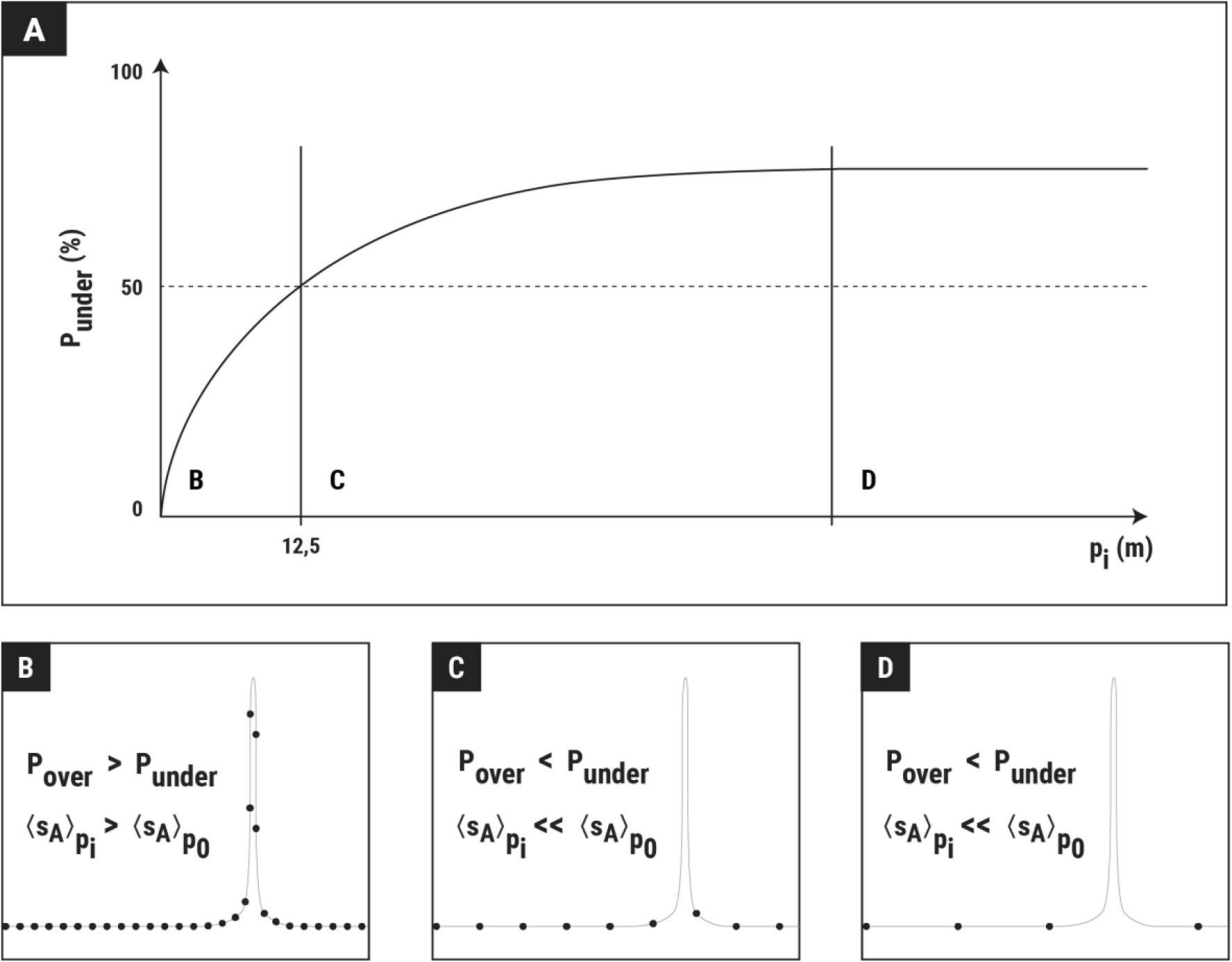
Graphical interpretation of the observed tendency of the underestimation probability (${P}_{under}$) with ping distance (${p}_{i}$). In the graph of the evolution of probability (**A**), three different parts can be distinguished depending on the ping distance range. For ping distances slightly greater than the original, the most likely missing pings are low values, slightly increasing the mean NASC and resulting in underestimation probabilities of less than 50% (**B**). As the ping distance increases, larger proportions of the transect samples are lost, increasing the probability of missing large values, and thus decreasing the mean abundance across replicates. At some point (~ 12.5 m in our case), the probability of overestimation outweighs the probability of underestimation (**C**). As this occurs due to the loss of some of the extremely large values, it coincides with a marked decrease in mean abundance. When the sampling resolution is very low, the probability of missing all rare large values is high, and the probability of underestimation tends to stabilize (as the mean NASC tends to a “mean without large values” (**D**)).

This behaviour can be explained by the skewed distribution of fisheries acoustic data, which contains many very small and a few extremely large values (Fig. S3). In this type of data distribution, increasingly low-resolution sampling leads to an increasing probability of missing some of the infrequent extreme values that dominate the overall abundance of the data. This has the direct consequence of the large difference between the mean NASC of resampling events that detect extreme values and those that do not, leading to an increase in uncertainty. In addition, increased low-resolution sampling results in increased dispersion and asymmetry of the dispersed values (Fig. 6), thus creating patterns in the deviation from the true values. The probability of overestimation outweighs the probability of underestimation at the lower end of the ping distances (Fig. 7). This occurs because the number of low values is much higher than that of high values, so if only a few pings are missing, it is more likely that one of the more frequent small values will be removed, resulting in slightly inflated averages compared to the true mean (Fig. 8). However, as the number of pings removed increases, so does the likelihood of missing some of the extreme values, increasing the likelihood of underestimation. Beyond a certain ping distance, the proportion of underestimates exceeds the proportion of overestimates and, finally, when the number of removed pings is large enough, the high probability of missing all the extremes causes the probability of underestimation to flatten out.

However, if missing extreme values favours underestimation, one might wonder why the mean deviation is zero, even in the worst resolution sampling scheme. In other words, why there is no contradiction between Fig. 7B and  C (mean and modal bias, respectively). The reason for this lies in a pattern exhibited by the asymmetric distribution of the resampled NASC values (Fig. 6). With very poor sampling resolution, in the rare occasions where extreme values are detected, the biomass is strongly overestimated (even reaching overestimations higher than 1500% in the top-right part of Fig. 4). When the survey is repeated many times (or simulated by resampling techniques), these rare and extreme cases of overestimation tend to compensate for the more frequent but smaller cases of underestimation, resulting in zero bias. Therefore, if a survey could be repeated many times, the mean of the abundances after many repetitions should be unbiased regardless of the sampling intensity, i.e., in the “long term” the abundance should be unbiased according to the Law of Large Numbers. However, this is not usually the case; although there may be exceptions when the study area is small^42^, there is usually only one opportunity to conduct the acoustic sampling, due to budget and time constraints^43^. In the case of a unique opportunity to sample the area, the most likely outcome of a poor sampling is a somewhat underestimated abundance, that is, in the “short term”, the poorly sampled abundance tends to be underestimated. Furthermore, the trend in the modal deviation from the resampled values shows that the most likely underestimation level increases with ping distance.

Abundance estimation therefore relies on the detection of extreme values, which, due to their scarcity, act as a population of “rare species”. For these rare populations, abundance estimates can be distorted by their low detection probability, often leading to biased estimates^44,45^. It should be noted that these typical rare extreme values found in acoustic data are not outliers, but rather perfectly valid values that occur naturally in any fishery campaign due to the contagious nature of fish populations^25^. The skewed nature of the acoustic data observed in this study is well known by the fisheries acoustics community and consistent with previous observations of fish density data^3,17^.

#### Effect of heterogeneity and autocorrelation

Two explanatory variables, heterogeneity and spatial autocorrelation, were included to assess their influence on the increase in uncertainty at low resolution. Heterogeneity was measured using inequality metrics, in particular the Gini index. This variable was used to provide information on the importance of extreme values in a given dataset. The results of the resampling exercise showed that the degree of heterogeneity of the acoustic data had a significant effect on the relationship between both random and systematic errors and ping resolution (Table S3 and Fig. 7). On the one hand, the increase in uncertainty with ping distance was steeper for higher levels of heterogeneity (Figs. 5 and  7). On the other hand, the most likely underestimation level (as indicated by the modal deviation) increased with heterogeneity.

The second categorical variable used in this study, spatial autocorrelation, is in the opposite direction of heterogeneity. The higher the spatial autocorrelation of the data, the slower the expected increase of CV, modal deviation, and probability of underestimation with the ping distance, with its effect being more pronounced at higher ping distances and levels of heterogeneity (Fig. 7). This means that, for example, in a heterogeneous and autocorrelated transect, extreme values would tend to cluster, thus increasing their likelihood of being detected and accounted for, even at poor sampling resolutions. Conversely, in a heterogeneous but uncorrelated transect, extreme values would be more dispersed along the transect, reducing their detectability at reduced resolution. This empirical result is consistent with the general geostatistical theory, which predicts lower variance in spatially autocorrelated datasets, unless the size of the region is large relative to the range of correlation^30^. This was accounted for in the definition of the spatial autocorrelation index, which includes not only the sill proportion but also the range proportion in its calculation (Eq. 13).

Some correspondence was observed between the fish aggregation typology in the echograms and the levels of heterogeneity and autocorrelation. For example, homogeneous layers tended to produce low levels of heterogeneity and correlation (Fig. S8-a). Heterogeneous layers and areas of scattered fish produced medium levels of heterogeneity and low or medium correlation (Fig. S8-b). Spatially extended, strong aggregations (i.e., persistent in most of the pings of the EDSU) produced high levels of heterogeneity and medium correlation; and strong, isolated schools produced both high heterogeneity and correlation levels (Fig. S8-c). Note, however, that these general patterns are difficult to summarise and are subject to frequent exceptions, as in many cases there may be a combination of several of the typologies in the same transect segment, and the degree of heterogeneity and correlation may depend on a single ping in the entire transect.

In early versions of the analysis, the influence of other ancillary variables on the evolution of errors with the ping interval was also explored, using the common morphological characteristics of the acoustic aggregations in the echograms^32^ obtained by school processing techniques^33–35^. The most significant of these morphological variables were the mean school area and occupation rate, which tended to slow down the increase in CV and modal deviation with ping distance, and the mean NASC and inter-school distance, which tended to accelerate it (Table S3). Other characteristics, such as the mean school depth, also slowed the error increase, but had less significant effect. However, as mentioned above, due to their lower explanatory power and to simplify the presentation of the main findings, this study focused on the effect of heterogeneity and spatial autocorrelation.

### Applicability of these results to other acoustic surveys

This analysis was based on acoustic data collected during a real trawl-acoustic survey^19^. The statistical properties of the acoustic data were consistent with those of previously published studies^17^. The systematic (or sequential) resampling method applied is believed to have reproduced the way in which the ping resolution is reduced in real acoustic surveys by increasing the ping interval or vessel speed, thus preserving the spatial autocorrelation of the fisheries acoustic data. The method used to obtain the averages per transect was consistent with that used in real trawl-acoustic surveys by including the intermediate step of calculating the mean abundances per EDSU (and even considering different EDSU values). Consequently, the understanding gained of the effect of reducing the along-transect resolution on precision and bias should be reliable and generally applicable to many real acoustic surveys (and, to some extent, to any other sampling method).

The resampling scheme applied, including the previous interpolation step (Fig. 2), allowed for well-balanced resampling sets with the same number of replicates for each ping resolution. The fact that the obtained mean abundance deviations were zero (Fig. 7) indicates that the number of replicates was sufficiently large to comply with the Law of Large Numbers. Alternatively, omitting the interpolation step would have resulted in a rigid sequential resampling scheme in which the maximum number of replicates would have been reduced, especially at the lowest and highest ping distances, with the resulting unbalanced resampling sets potentially distorting the obtained uncertainty and bias tendencies with resolution. This occurs because, when relying solely on the available pings to perform sequential resampling (i.e., remove every second ping, every third ping, etc…), the available combinations at each resolution are finite, particularly at the highest and lowest ping distances. For instance, in the resampling corresponding to the highest resolution (lowest ping distance), one ping must be removed in each EDSU. This leaves as many different resamples as there are pings in the EDSU, in the order of 400–1000 possible repetitions according to the ping distances of this work (considerably less than the 3000 repetitions established thanks to the interpolation step). The number of combinations is exactly the same for the lowest resolution (highest ping distance), where one ping in each EDSU must be kept, again giving 400–1000 different resamples. Note that this problem is exacerbated when using smaller EDSU lengths or higher initial ping distances.

This analysis was carried out at the level of transects or transect segments of approximately 23 km in mean length, each assigned to a single species composition. Therefore, if applied to an acoustic survey where species attribution is performed using a “reference haul” approach^3^, the application should be straightforward for each transect segment associated with a given reference haul. This could also be applied to larger homogenous strata containing many transects or to entire surveys, as the general trends would be valid, and it could be useful to compare different surveys or make interannual comparisons of the same survey. However, the estimated errors should be considered as relative rather than absolute (as will be discussed below).

Since the Gini index increases with the ping distance (Fig. S9), when applying the results of this study to a real survey conducted at a lower ping resolution, the heterogeneity measured at higher ping distances should be reduced to that of the original data before estimating the expected errors. However, since a correction of 0.6% in the Gini index value should be applied for each 5 m increment in the ping distance (Fig. S9), the correction is barely noticeable in practice. For example, for a transect sampled with an average ping distance of 17.5 m, where a Gini value of 0.7 has been estimated, there is an increase in ping distance of ~ 15 m with respect to the reference; therefore, the Gini should be reduced by 1.8%, i.e. to 0.69, which leaves us with practically the same heterogeneity scenario.

There are some other considerations in this study that should be noted. Perhaps the most important limitation is the large variability in the resampling results. Even though the trends in uncertainty and bias were clear and stable across the different transects, there was a large variability in the trends obtained, probably in part because of the different values registered on each transect (Figs. 5 and  8). This means, for example, that the bias values will depend not only on the level of heterogeneity and autocorrelation, but also on the actual extreme values and how much they influence the mean values, which do not seem to be fully captured in the indices.

This can also be applied to the categorical variables used. Although heterogeneity and autocorrelation levels generally control the rate of precision loss and underestimation, when more than three categories are included, there may be inverted or constant trends in the intermediate values (not shown). Therefore, heterogeneity indices improve predictions (Table S3), especially when applied to acoustic data with clear contrasts. Where small differences exist, they do not always translate into proportional differences in error change. This may be due to the relatively small number of transects (4–6) within each range of categorical values. It is likely that the results would have been more consistent if a larger number of transects had been included in the analysis.

The acoustic survey used as the basis for the resampling analysis was recorded using a 7° beamwidth transducer. Therefore, strictly speaking, the results obtained are only valid for this type of transducer. However, in an attempt to provide some insight into the potential effect of different beamwidths on the results, the sampled fraction of the along-transect plane (Fig. 1) was calculated for different combinations of ping distances and transducer beamwidths. Since, according to Eq. 17, an increase in transducer bandwidth increases the sampled fraction for the same ping distance, it is expected to decrease the probability of underestimation (shifting the plots in Fig. S10-A to the right). Consequently, increasing the transducer beamwidth tends to increase the threshold of safe ping distances (i.e., the maximum ping distances at which the probability of underestimation is low; Fig. S10-B). It is likely, however, that this reduction in the risk of bias would be at the expense of a loss in precision due to the greater variability in acoustic backscattering as a result of the increase in sampling volume (greater variability in the tilt angles of detected targets, lower signal-to-noise ratio of these types of transducers, etc.).

This study only tested the purely statistical effect of a reduced sampling rate on the average acoustic backscatter. It was not measured the potential incidence of the reduced resolution on the appearance of acoustic aggregations on the echograms, and the potential effect this might have had on the assessment through the scrutiny process.

This study focused on studying a survey applying trawl-acoustic methodology using vertical transducers installed on a mobile platform traversing an area. Some of the assumptions associated with this methodology entail sampling at a speed higher than the average swimming speed of the target species. Therefore, potential biases due to non-compliance with this requirement were not addressed in this investigation. Furthermore, the analysis carried out was completely spatially oriented and the considerations derived from the study pertain to the spatial resolution of the sampling within the target area. Consequently, deducing the possible consequences of increasing the ping interval for a stationary platform would be challenging and out of the scope of this study, as it would involve discussing temporal rather than spatial sampling and resolution, subject to a completely different set of assumptions.

Finally, this study assumes that the abundance estimate obtained using the original, minimum ping-distance resolution is accurate. The average ping distance used in the survey was 2.65 m and ranged from 1.05 m to 3.7 m, with standard deviation of 0.85 m. The sampling intensity used is consistent with that typically applied in acoustic-based assessment surveys^8^; therefore, even if it was not possible to cover the full range of possible ping distances, the study should be able to reflect and inform about typical and realistic conditions encountered in actual acoustic surveys.

Based on these considerations, the main value of this study lies in its explanatory power. It should be reliable in describing the general effect of poor along-transect sampling on the error of abundance estimates, but the predictions of the actual errors should be considered relative, as the level of increased dispersion and underestimation will depend on the particular level of the large values detected. However, even if the predicted error levels are relative, the general trends observed are robust. It is therefore worth noting the change in trend that occurs at ping intervals of ~ 12.5 m for any category of heterogeneity and autocorrelation. Below this interval, although the simulation predicts slightly overestimated results, the level of overestimation is generally close to zero. Beyond ~ 12.5 m, however, the predictions are often underestimated, and here the level of underestimation becomes noticeable. Therefore, this ~ 12.5 m interval could be taken as an easy-to-remember safety threshold, beyond which areas of potentially biased abundance estimation are reached. Considering the typical speed of sound in seawater (and ignoring possible processing or synchronisation delays in the acoustic equipment), this would lead to maximum ranges of over 1800 m, which is sufficient to completely cover the entire epi- and meso-pelagic zones (i.e., down to 1000 m depth).

The origin of this analysis was the concern about the increase in the maximum sampling distance in the JUVENA campaign. In this campaign, starting in 2015, the recording range was increased from 250 to 500 m in order to estimate the abundance of pearlside (*Maurolicus muelleri*), the most abundant and accessible mesopelagic species in the Bay of Biscay. Based on the results obtained, the applied increase in recording range resulted in a longer ping interval (of approximately 1 s), resulting in ping distances of approximately 5 m, which placed the along-transect resolution well within the safety margin described above. Consequently, the campaign was continued with the extended ping interval without any additional measures.

## Conclusions

The consequences of a reduced resolution along the transect for acoustic-based abundances are an increase in uncertainty and an increase in the probability and magnitude of underestimation caused by the highly skewed distribution of acoustic data, where the mean values are heavily influenced by a few extreme values. High heterogeneity and low spatial autocorrelation accelerate the increase in uncertainty and underestimation levels with decreasing resolution.

### Supplementary Information

Supplementary Information.

## Data Availability

The data underlying this article will be shared on request to the corresponding author.
