# Supplementary material for: Understanding the impact of along-transect resolution on acoustic surveys
Source: Sci Rep. 2023 Aug 22;13:13687. doi: 10.1038/s41598-023-40960-6 (PMC10444787; doi:10.1038/s41598-023-40960-6)
Supplement: Supplementary file 1 — Supplementary Information. [file 41598_2023_40960_MOESM1_ESM.docx]

**Supplementary Information**


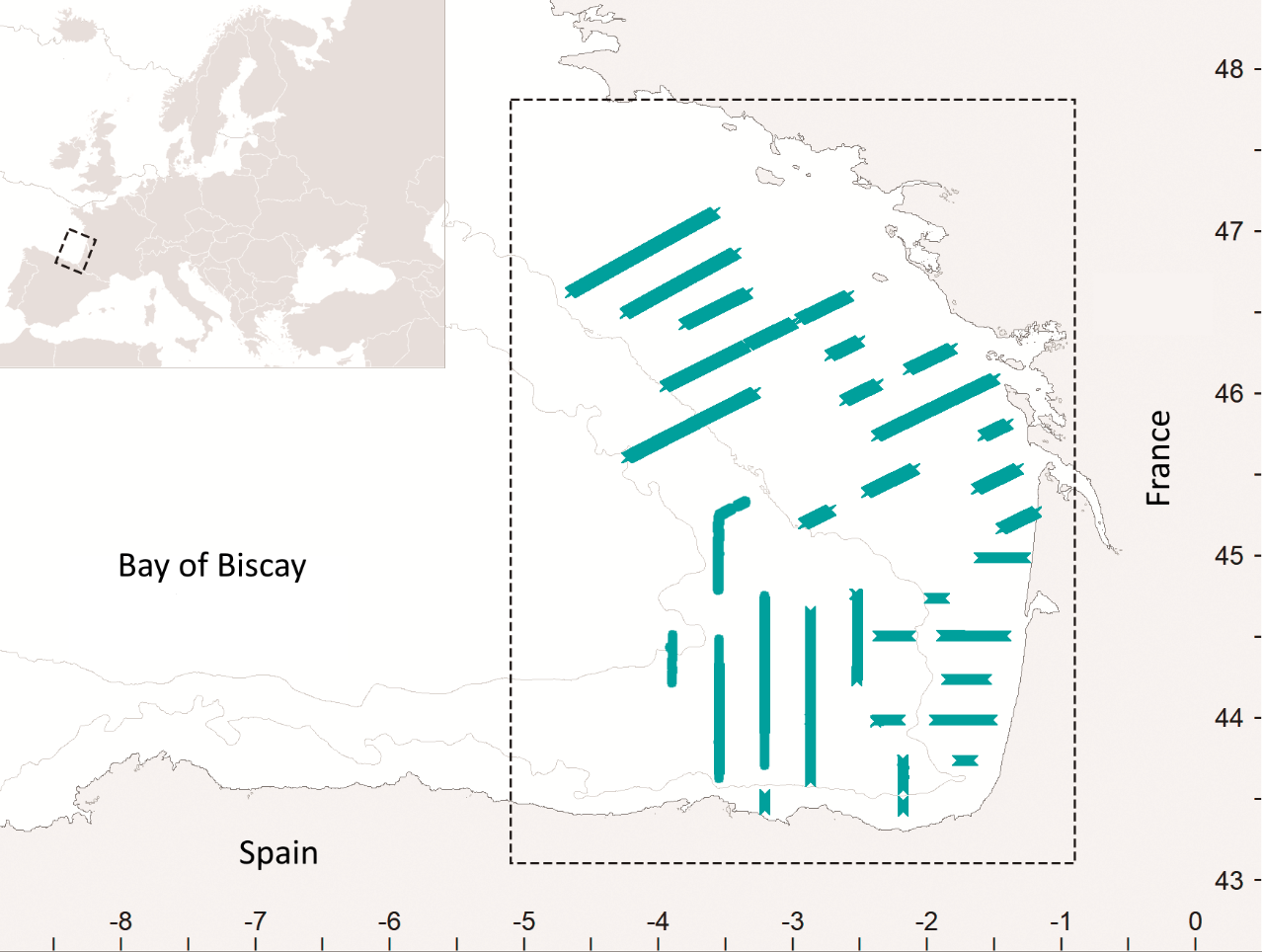


Figure S1. Segments of acoustic transects from JUVENA 2010 on which the resampling scheme was applied.


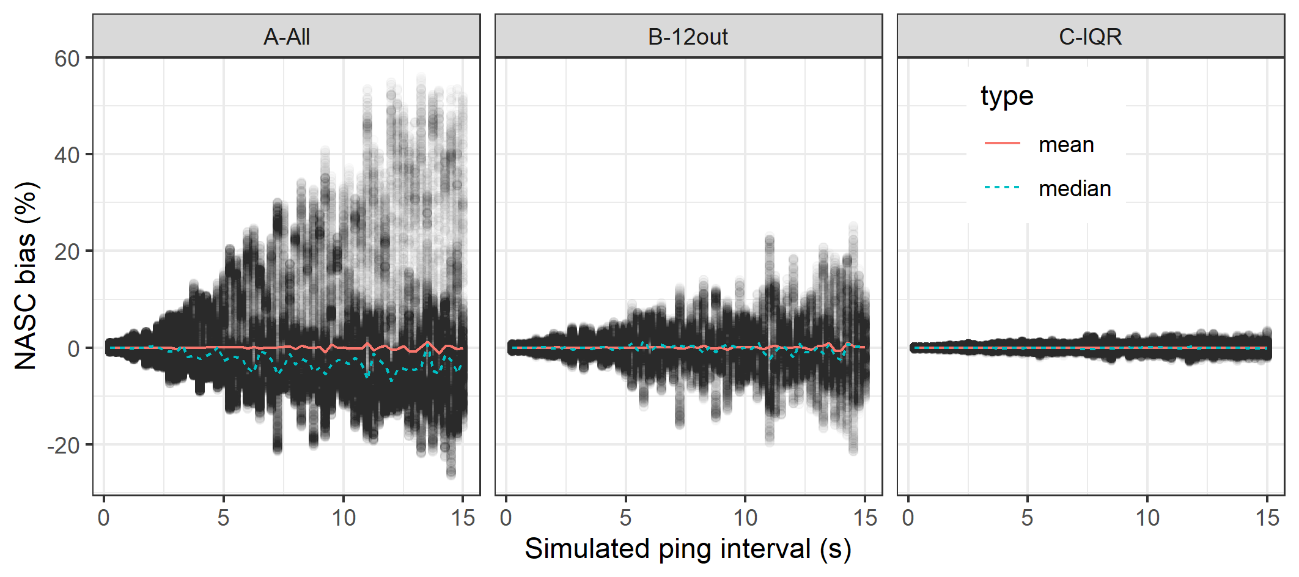


Figure S2. Scatterplot of mean resampled NASC bias as a function of simulated ping interval in a resampling exercise conducted in a single stratum to analyze the effect of extreme values (“outliers”). The lines (colored in the online version) show the mean and median of the mean resampled NASC biases at each pint interval. (A) Graph done using all the data. The spread of the points shows a clear increase of uncertainty with ping interval. The mean values remain constant, but the decrease of the median values shows the increase of underestimation bias with increasing ping interval. (B) The same graph showing the result of the analysis after removing the highest 12 values (0.011% of the data). The uncertainty increases with ping interval at a slower pace, and the decrease of the median (as an indication of the likelihood of underestimation bias) is highly reduced. (C) The same graph after removing outliers according to the IQR criterion. The uncertainty still increases with ping interval, although at a considerably slower pace than the other two cases, and the bias is practically removed. For details on this resample analysis see the preprint of an earlier version of this study in the following link:

<https://www.researchgate.net/publication/365002487_Impact_of_ping_interval_and_data_heterogeneity_on_acoustically_based_abundances>.


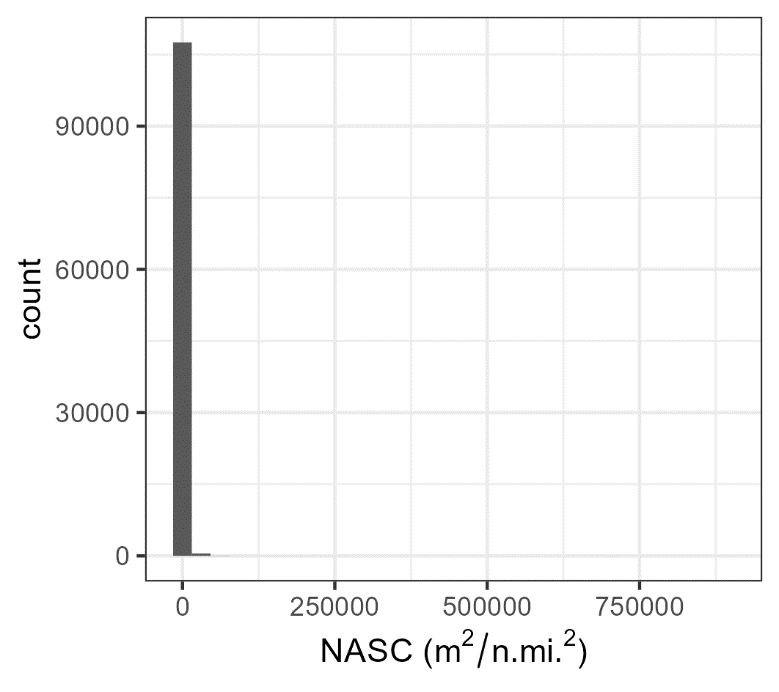


Figure S3. Histogram showing the statistical distribution of the data corresponding to the single stratum resampling analysis referred to in Figure S2. The figure shows the skewed nature of fisheries acoustics data, with the great majority of values close to zero and a few extremely large ones that dominate the mean of the distribution.


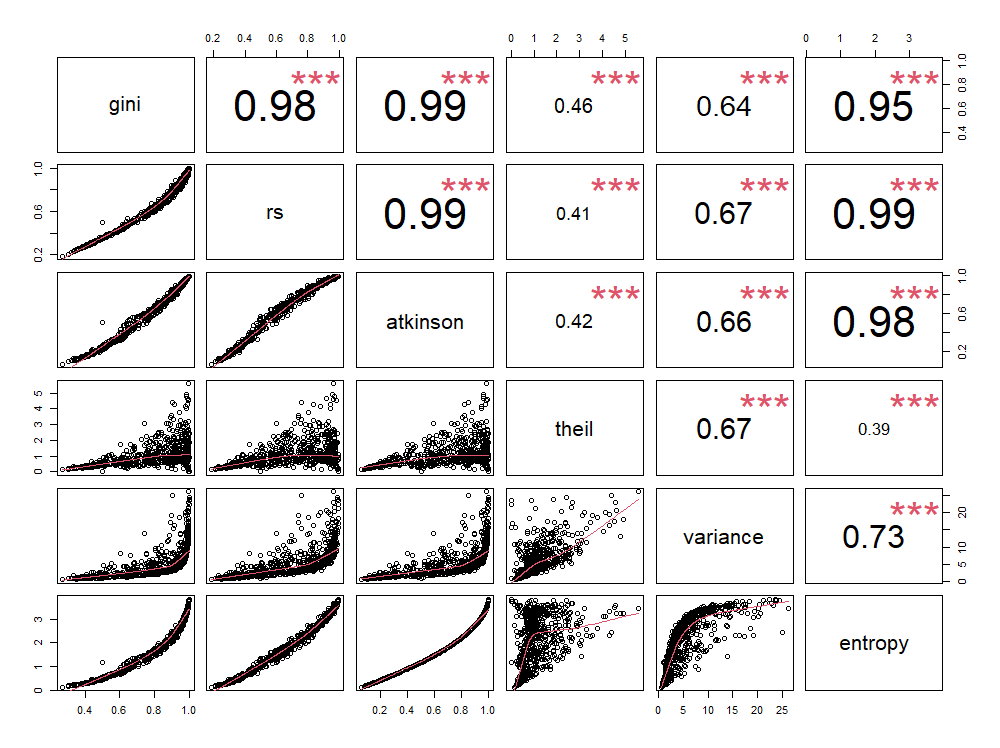


Figure S4. Pair-plot showing correlation between pairs of heterogeneity indices. The numbers mark the correlation coefficient plus the significance (*** meaning highly significant correlation between all of them). The lower-left panels present scatterplots between the variables with smoothers. Note: variance stands for standard deviation.


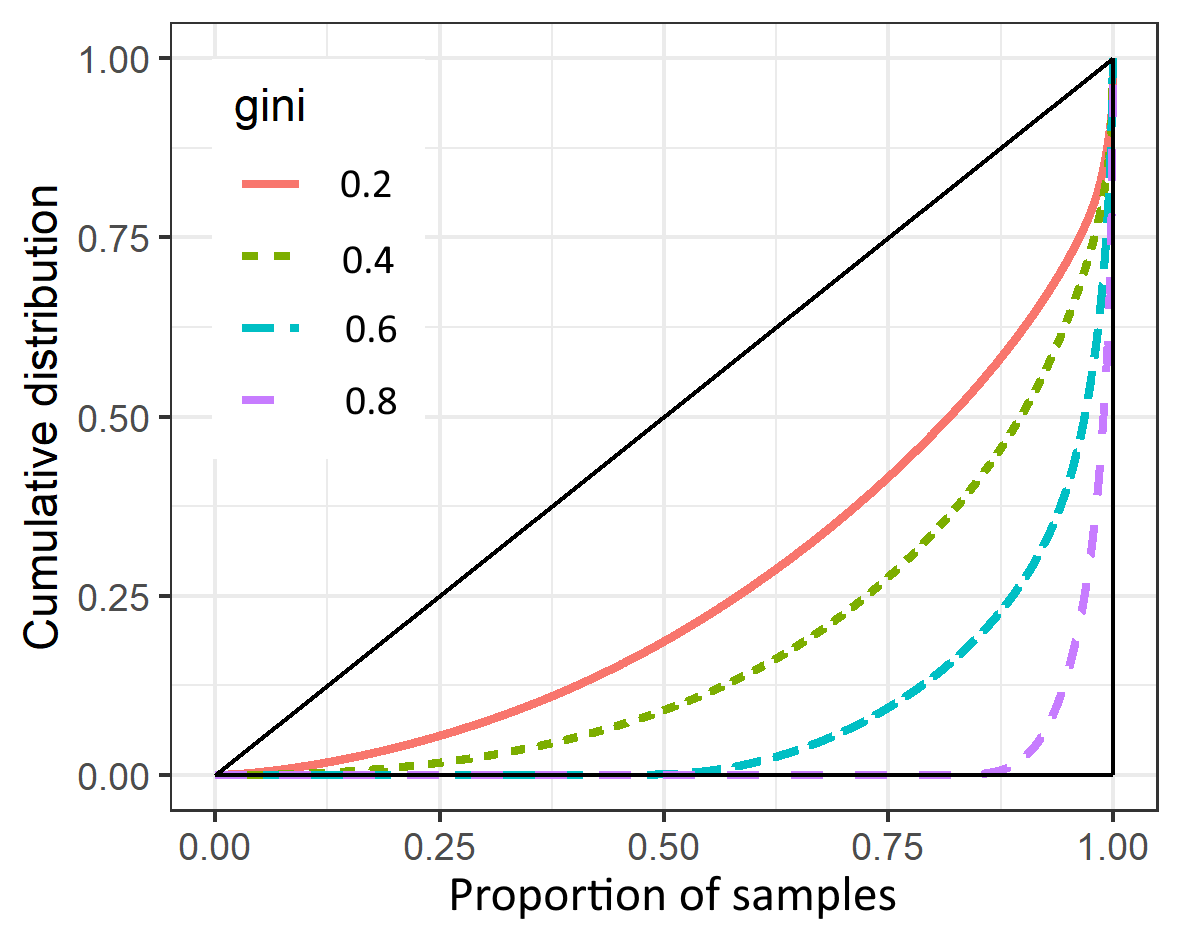


Figure S5. Example of Lorenz curves (curved lines, coloured online) for some of the real transects used in the analysis, each with different values of heterogeneity given by the Gini index. The curves represent the cumulative distribution of ordered abundance in relative terms against the proportion of samples in the transect. The Gini index of inequality is obtained by comparing the area under the diagonal and the area under each of the Lorenz curves. Smoothly increasing curves close to the diagonal have large areas and represent homogeneous (low Gini) transects, whereas curves with a long proportion of zeroes followed by a steep increase of the slope have small areas and represent heterogenous (high Gini) transects.


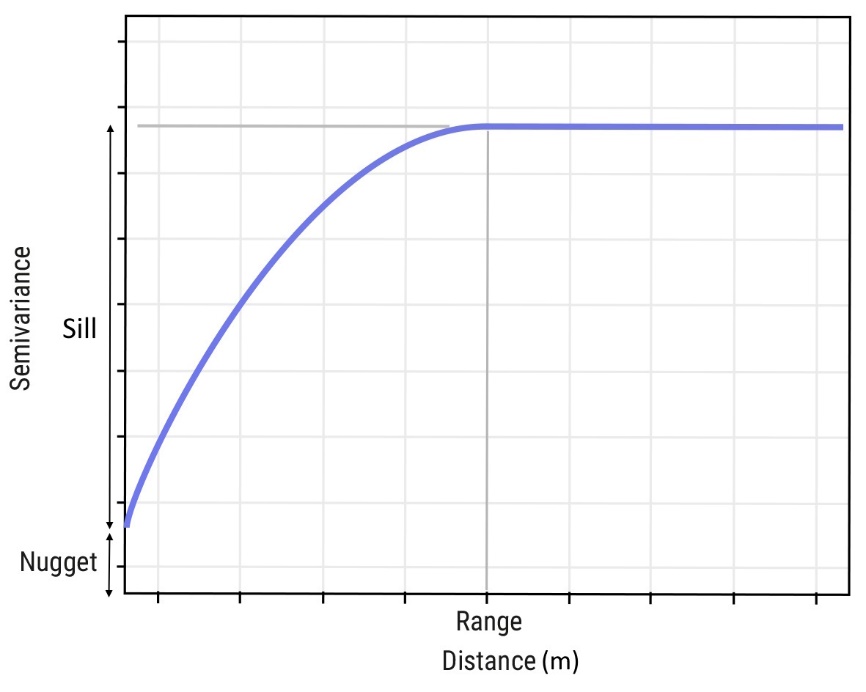


Figure S6. Illustration of a generic variogram, a function describing the spatial autocorrelation of georeferenced data. The curve describes the evolution of the correlation between pairs of data as a function of their mutual distance, and is used to obtain the features that summarise the spatial autocorrelation of the data: sill, nugget and range.


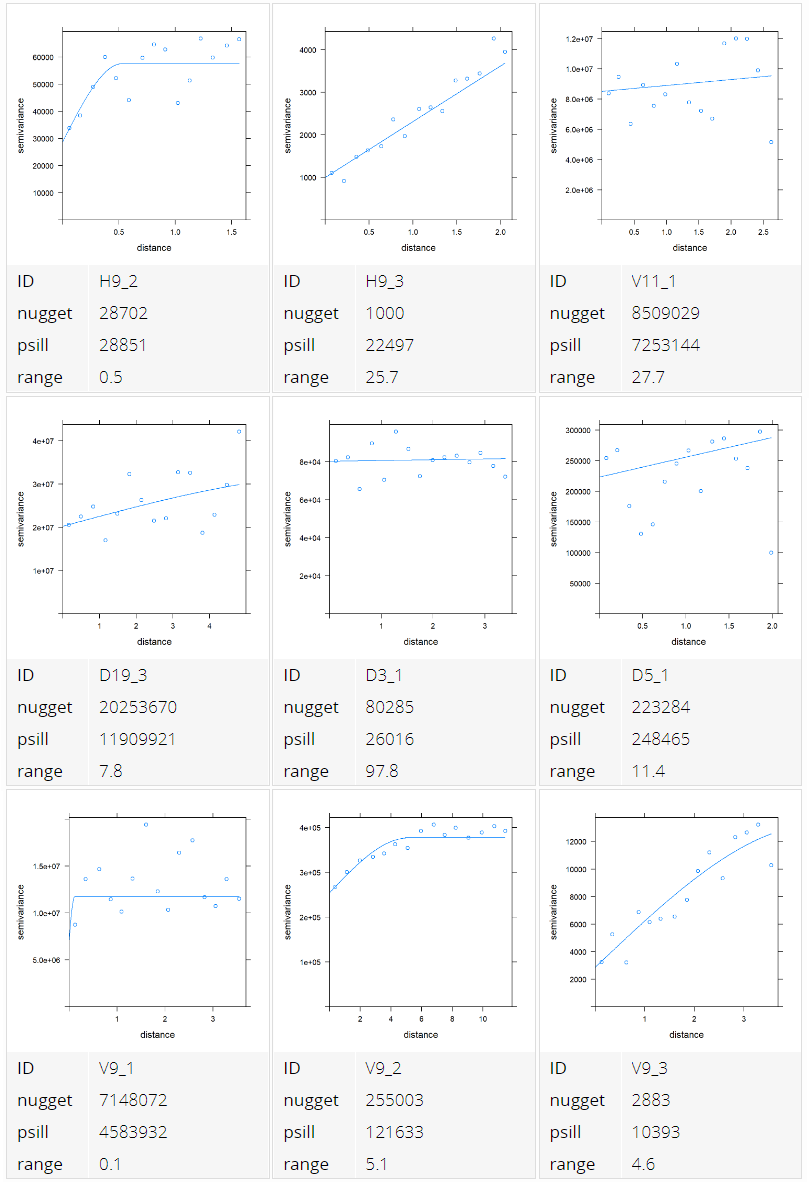


Figure S7. Examples of experimental semivariograms of a sample of transects from the empirical acoustic data, showing the nugget, sill and range values extracted from each variogram. The examples were chosen to cover a range of different autocorrelation levels, low (D3_1, V9_1, D19_3), medium (H9_2, V9_2, D5_1, V11_1) and high (V9_3, H9_3).


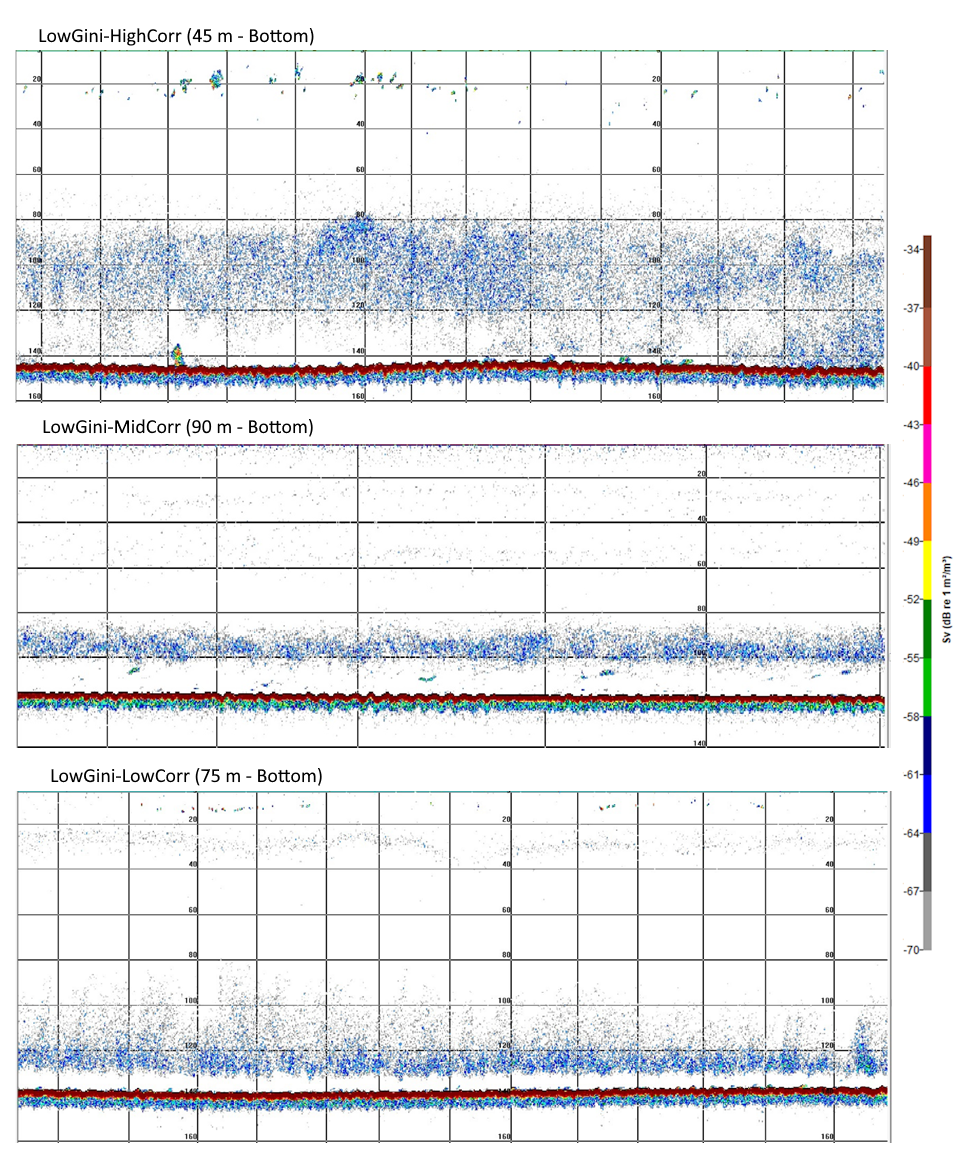


Figure S8-a. Examples of echograms with low heterogeneity and different levels of spatial correlation at the -70 dB threshold. The minimum and maximum echo integration depths are given in brackets. The vertical lines mark 0.1 nmi (185.2 m). Note that the seafloor is excluded from echointegration and does not contribute to heterogeneity or autocorrelation.


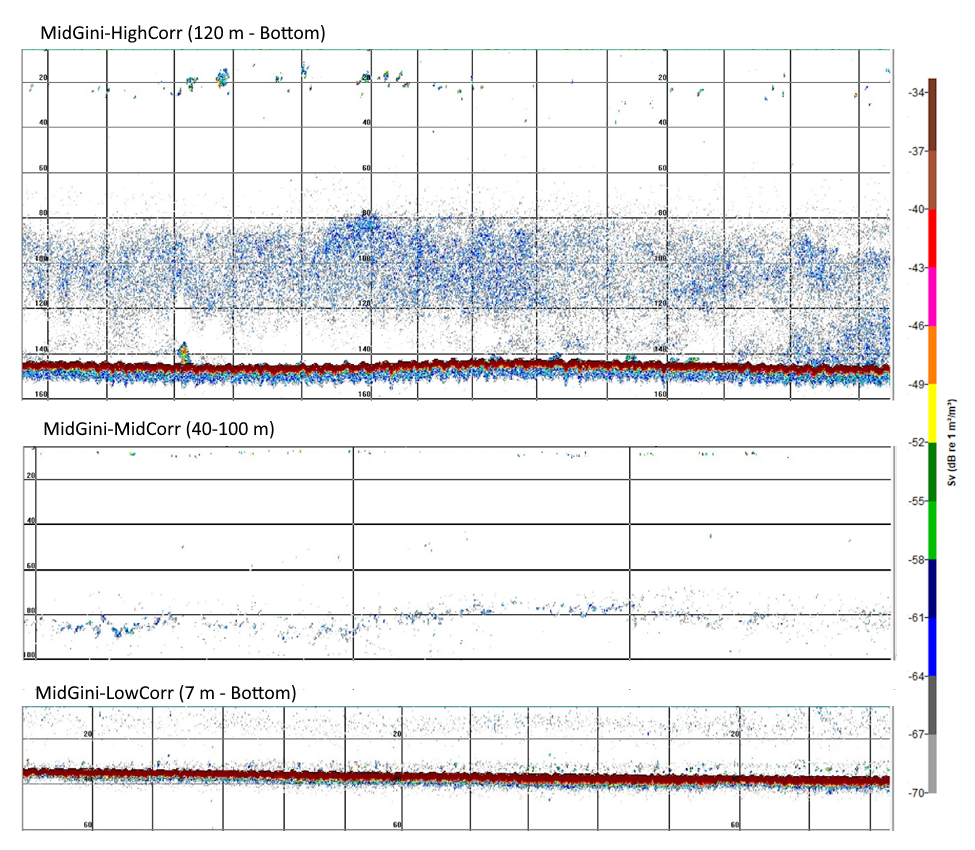


Figure S8-b. Examples of echograms with medium heterogeneity and different levels of spatial correlation at the -70 dB threshold. The minimum and maximum echo integration depths are given in brackets. The vertical lines mark 0.1 nmi (185.2 m). Note that the seafloor is excluded from echointegration and does not contribute to heterogeneity or autocorrelation.


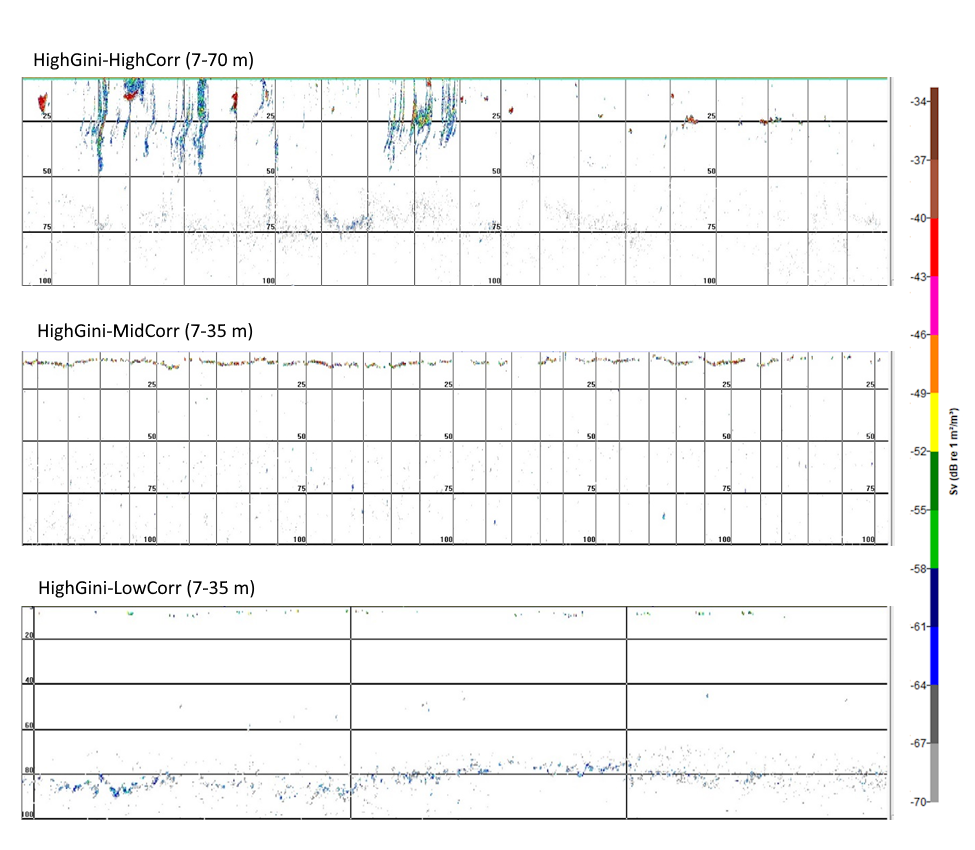


Figure S8-c. Examples of echograms with high heterogeneity and different levels of spatial correlation at the -70 dB threshold. The minimum and maximum echo integration depths are given in brackets. The vertical lines mark 0.1 nmi (185.2 m).


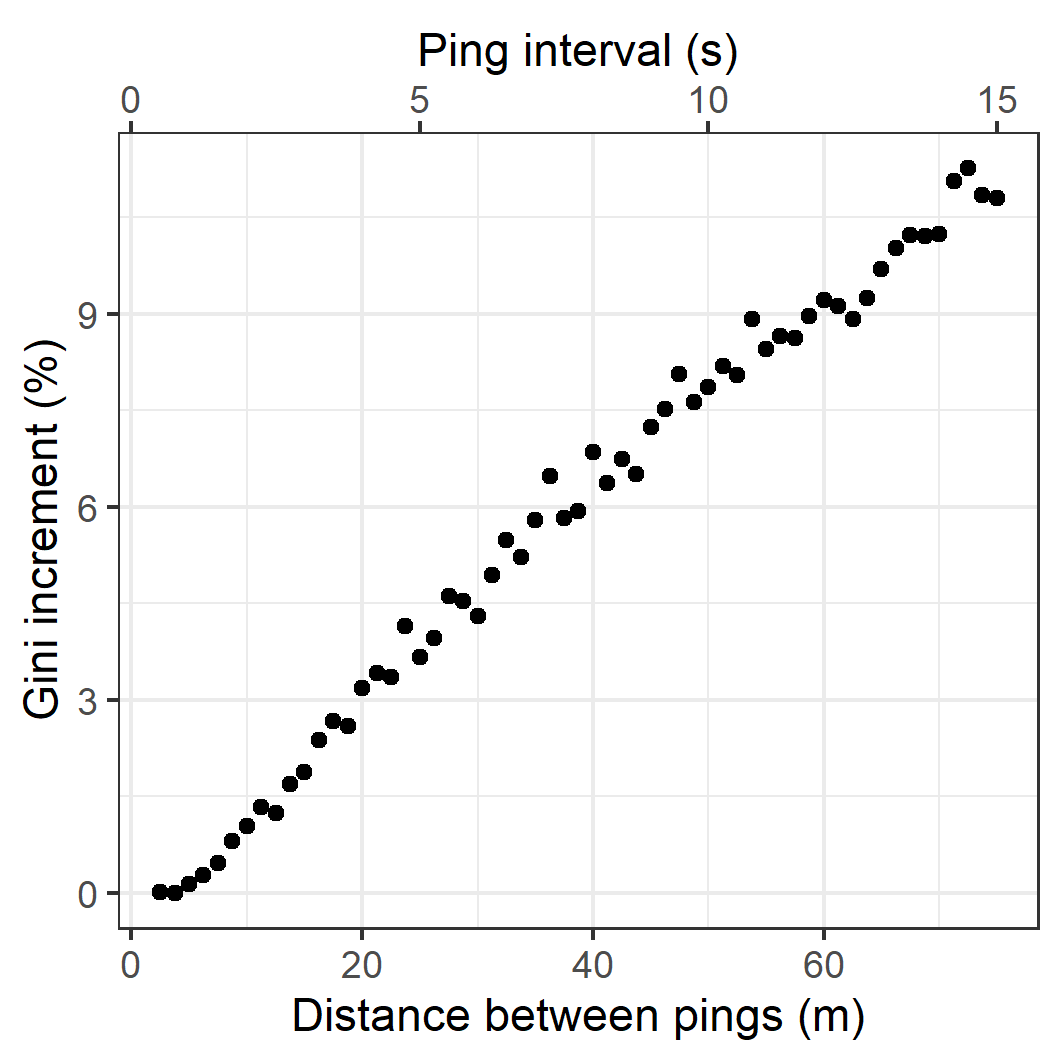


Figure S9. Increase in the Gini index with the ping distance, provided as a percentage with respect to the index at the original ~2.5 m distance.


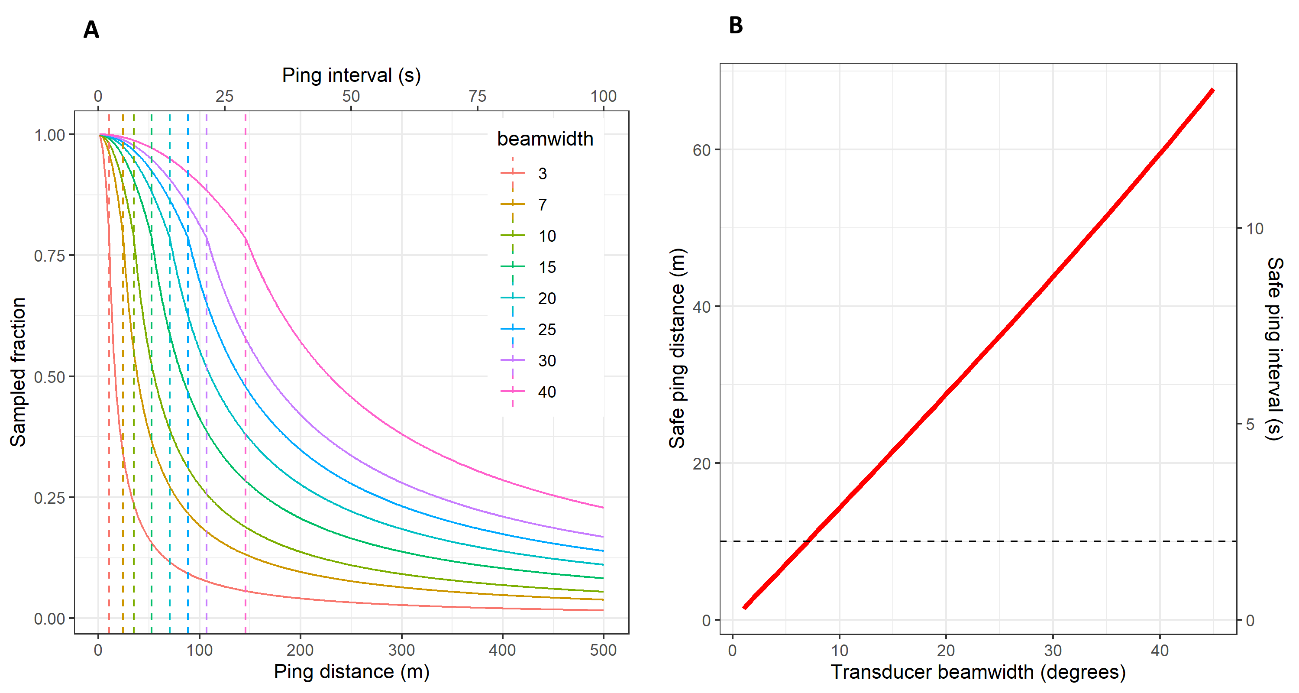


Figure S10. (A) Relationship between along-transect sampled fraction and pinging resolution for different common transducer beamwidths (in degrees). As the transducer beamwidth increases, the sampled fraction increases for the same ping distance, thus making it less likely to be biased. (B) As a result of the increased sample fraction with transducer beamwidth, the "safe” ping resolution (maximum ping distance at which the risk of underestimation is low, established at ~12.5 m for a 7° beamwidth, marked with a horizontal dashed line) increases with wider angle transducers.

Table S1: List of the parameters of the school processing process applied with Movies+ to estimate aggregation typologies of the echotraces.

| **Parameter** | **Value** | **Units** |
| --- | --- | --- |
| Minimum $S_{V}$ threshold | -60 | dB |
| Maximum $S_{V}$ threshold | -10 | dB |
| Queue threshold | -20 | dB |
| Min energy ($\sigma_{ag}$) | 0.0001 | m^2^ |
| Max energy ($\sigma_{ag}$) | 100 | m^2^ |
| Min height | 10 | m |
| Max height | 1000 | m |
| Min length | 10 | m |
| Max length | 1000 | m |
| Min area | 5 | m^2^ |
| Max area | 100000 | m^2^ |
| Min density ($S_{V}$) | -120 | dB |
| Max density ($S_{V}$) | 0 | dB |
| Initial number | 1 |  |
| Horizontal gap | 0 | pings |
| Vertical gap | 0 | m |
| Horizontal tolerance | 5 | pings |
| Vertical tolerance | 3 | m |

Table S2: List of the most significant characteristics of schools or aggregations used as auxiliary variables in the preliminary model prediction analysis. All the characteristics are computed as averages per transect. From this list, after Variance Inflation Factor analysis and removal of the least significant, the selected variables were NASC, Dist, Area and OccRate.

| **Characteristic** | **Units** | **Description** |
| --- | --- | --- |
| *Nshoal* |  | Number of aggregations |
| *NASC* | m^2^/nmi^2^ | Nautical Area Scattering Coefficient |
| *MVBS* | dB re 1 m^-1^ | Mean Volume Backscattering Strength - $\left\langle S_{V} \right\rangle$ |
| *Depth* | m | Depth |
| *Length* | m | Length |
| *Height* | m | Height |
| *Area* | m^2^ | Area |
| *Perim* | m | Perimeter |
| *Vol* | m^3^ | Volume |
| *Elong* | m/m | Elongation: $Length/Height$ |
| *FD* | log(m)/log(m) | Fractal dimension: $2\log\left( {Perim}/4 \right)/log(Area)$ |
| *Dist* | m | Mean distance between aggregations |
| *OccRate* | % | Occupation rate |

Table S3: Model predictions of uncertainty (CV) and modal deviation (MedianDev) and probability of underestimation against ping distance (*p_i_*), a quantitative (Quantit.) variable, plus the full set of selected categorical (Categ.) auxiliary variables. Linear and GAM models were compared using AIC. Asterisks indicate the approximate significance of explanatory variables (p-values: “***” < 0.001; “**” < 0.01; “*” < 0.05 and “ ” < 1)

|  | **LM** | | | | **GAM** | | | |
| --- | --- | --- | --- | --- | --- | --- | --- | --- |
| **Dep.** | **Quantit.** | **Categ.** | **Categ.** | **AIC** | **Quantit.** | **Categ.** | **Categ.** | **AIC** |
| CV | *p_i_**** |  |  | 31844 | *p_i_**** |  |  | 31787 |
|  | *p_i_**** | Gini*** |  | 31520 | *p_i_**** | Gini*** |  | 31454 |
|  | *p_i_**** | Corr*** |  | 31575 | *p_i_**** | Corr*** |  | 31524 |
|  | *p_i_**** | OccRate*** |  | 31756 | *p_i_**** | OccRate*** |  | 31737 |
|  | *p_i_**** | Area*** |  | 31729 | *p_i_**** | Area*** |  | 31580 |
|  | *p_i_**** | NASC** |  | 31765 | *p_i_**** | NASC** |  | 31753 |
|  | *p_i_**** | Dist* |  | 31839 | *p_i_**** | Dist* |  | 31837 |
|  | *p_i_**** | Gini*** | Corr*** | 31219 | *p_i_**** | Gini*** | Corr** | 31095 |
| ModalDev | *p_i_**** |  |  | 28089 | *p_i_**** |  |  | 28084 |
|  | *p_i_**** | Gini*** |  | 27881 | *p_i_**** | Gini*** |  | 27871 |
|  | *p_i_**** | Corr*** |  | 27938 | *p_i_**** | Corr*** |  | 27927 |
|  | *p_i_**** | OccRate*** |  | 28037 | *p_i_**** | OccRate*** |  | 28030 |
|  | *p_i_**** | Area*** |  | 28016 | *p_i_**** | Area*** |  | 27871 |
|  | *p_i_**** | NASC* |  | 28075 | *p_i_**** | NASC** |  | 28074 |
|  | *p_i_**** | Dist* |  | 28067 | *p_i_**** | Dist* |  | 28047 |
|  | *p_i_**** | Gini*** | Corr*** | 27719 | *p_i_**** | Gini*** | Corr*** | 27702 |
| P_under_ | *p_i_**** |  |  | -3082 | *p_i_**** |  |  | -3467 |
|  | *p_i_**** | Gini* |  | -3088 | *p_i_**** | Gini** |  | -3615 |
|  | *p_i_**** | Corr** |  | -3182 | *p_i_**** | Corr*** |  | -3639 |
|  | *p_i_**** | OccRate* |  | -3088 | *p_i_**** | OccRate* |  | -3615 |
|  | *p_i_**** | Area** |  | -3134 | *p_i_**** | Area*** |  | -3621 |
|  | *p_i_**** | NASC** |  | -3271 | *p_i_**** | NASC*** |  | -3627 |
|  | *p_i_**** | Dist |  | -3080 | *p_i_**** | Dist*** |  | -3631 |
|  | *p_i_**** | Gini* | Corr*** | -3189 | *p_i_**** | Gini* | Corr*** | -3648 |
